# Supplementary material for: Quantum molecular resonance and secretome synergistically unlocks a protective ceRNA axis in the human retinal pigment epithelium
Source: Mol Ther Nucleic Acids. 2026 Jul 22;37(3):103034. doi: 10.1016/j.omtn.2026.103034 (PMC13471074; doi:10.1016/j.omtn.2026.103034)
Supplement: Supplementary file 1 [file mmc1.pdf]

OMTN, Volume *37*

Supplemental information

Quantum molecular resonance and secretome synergistically unlocks a protective ceRNA axis in the human retinal pigment epithelium

Simona Alibrandi, Domenico Mordà, Concetta Scimone, Angela D'Ascola, Federica Aliquò, Giorgia Abate, Gianantonio Pozzato, Sergio Zaccaria Scalinci, Rosalia D'Angelo, Antonina Sidoti, and Luigi Donato

Supplemental Material

Supplemental Tables

Table S1. Complete differential expression statistics across six full-factorial experimental contrasts. This master table provides global differential expression profiling of all detected non-coding transcripts. For each of the six pairwise comparisons (QMR vs. CTRL, SECRETOME vs. CTRL, QMR+SEC vs. CTRL, QMR+SEC vs. QMR, QMR+SEC vs. SEC, and SEC vs. QMR), the table reports the corresponding $\log_2$ Fold Change ($\log_2$FC) and False Discovery Rate (FDR/adjusted p-value). Transcripts that met the rigorous significance thresholds (FDR < 0.05, $|\log_2FC| > 1$) were flagged for each specific contrast.

Provided as a separate Excel file: Table_S1_Differential_Expression_Complete.xlsx.

Table S2. Comprehensive inventory of the ARPE-19 non-coding transcriptome. Complete catalog of the 2,313 detected non-coding transcripts (TPM > 0.1) across all 72 biological samples. The dataset included Ensembl IDs, assigned gene symbols, exact genomic coordinates (chromosome, start, end, and strand), exon counts, and transcript biotype classifications (e.g., antisense, lincRNA, sense-intronic, and circRNA). Mean Transcripts Per Kilobase Million (TPM) values were provided for the CTRL, QMR, SECRETOME, and QMR+SECRETOME experimental groups to establish baseline expression levels.

Provided as a separate Excel file: Table_S2_All_Detected_lncRNAs.xlsx.

Table S3. Gene Ontology (GO) functional enrichment analysis of differentially expressed non-coding networks. The comprehensive output of the pathway enrichment analysis derived from the protein-coding targets strongly correlated with the master ncRNAs. The table categorizes significantly enriched terms (FDR < 0.05) into three primary GO ontologies: Biological Process (BP), Molecular Function (MF), and Cellular Component (CC). For each enriched term, the table details the GO ID, biological description, number of mapped genes, Gene Ratio, exact FDR-adjusted p-value, and specific gene set driving the enrichment (e.g., BMP2, BMP6, and FADS2).

Provided as a separate Excel file: Table_S3_GO_Enrichment_Complete.xlsx.

Table S4. Oligonucleotide primer sequences designed for the qRT-PCR validation of prioritized non-coding transcripts. The table reports the sequence features (Forward and Reverse primers, 5'→3' orientation) and expected amplicon size for the top 10 significantly modulated ncRNAs selected for technical validation. GAPDH and ACTB were used as internal endogenous reference controls for normalization. For circular RNAs, circASPH and circCRIM1, specifically designed divergent primers spanning the unique non-canonical back-splice junction were employed to prevent co-amplification of linear host transcripts.

Gene Symbol	Primer Direction (5'_3')	Sequence (5'_3')	Expected Amplicon Size (bp)
circASPH(divergent)	Forward	GCCAAGATCTGAGGCCTACC	148
	Reverse	CTCAGGTTCTTGGCCACATC	
circCRIM1(divergent)	Forward	TGTGTCCTGGCTACTGTGTC	135
	Reverse	ACAGCAGTAGCTCCCACATC	
NEAT1	Forward	TGGCTAGCTCAGGGCTTCAG	172
	Reverse	TCTCCTTGCCAAGCTTCCTTC	
BASP1-AS1	Forward	GGCATCCAGACACAGCATTC	155
	Reverse	CCCTGGAGCTCCAAACATCA	
MALAT1	Forward	AAAGCAAGGTCTCCCCACAAG	129
	Reverse	GGTCTGTGCTAGATCAAAAGGCA	
TAPBP-AS1	Forward	TCTGGCCCATGTCACAAAGT	160
	Reverse	GGCCTCAGCCTCTCTTTAGC	
LINC-ROR	Forward	AGGAAGCCTGAGAGTTGGC	185
	Reverse	CTCAGTGGGGAAGACTCCAG	
SOD2-OT1	Forward	GGACACGAAGAATCTGAGGC	142
	Reverse	CTTCAGCATTGCCCTTACCC	
RELA-DT	Forward	CCTGGGATCAGATGTGTGGT	150
	Reverse	TCTCAGCCCTGGATTTTGCG	
CSRNP1-AS1	Forward	AGCACAGAGCAGAAGTTGCT	165
	Reverse	TGGAGATGCGGCTGATTAGG	
GAPDH(Control)	Forward	GGAGCGAGATCCCTCCAAAAT	197
	Reverse	GGCTGTTGTCATACTTCTCATGG	
ACTB(Control)	Forward	CATGTACGTTGCTATCCAGGC	250
	Reverse	CTCCTTAATGTCACGCACGAT	

Supplemental Figures

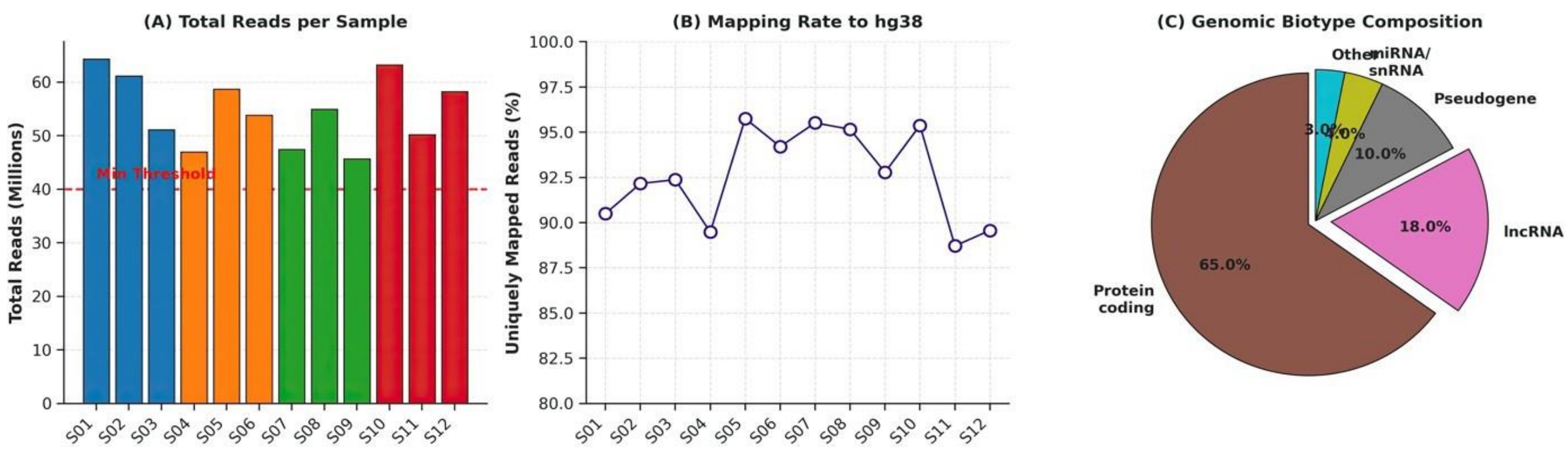

Figure S1. RNA-Seq Quality Control and Read Mapping Statistics. (A) Bar chart showing the total number of raw reads sequenced per sample across all biological replicates. The dashed red line indicates a minimum threshold of 30 million reads. (B) Box-and-whisker plots illustrating the percentage of high-quality reads uniquely mapped to the GRCh38/hg38 human reference genome for each sample. (C) Horizontal bar chart summarizing the total transcriptomic composition of ARPE-19 cells classified by primary genomic biotypes (e.g., protein-coding mRNAs, lncRNAs, pseudogenes, and small RNAs).

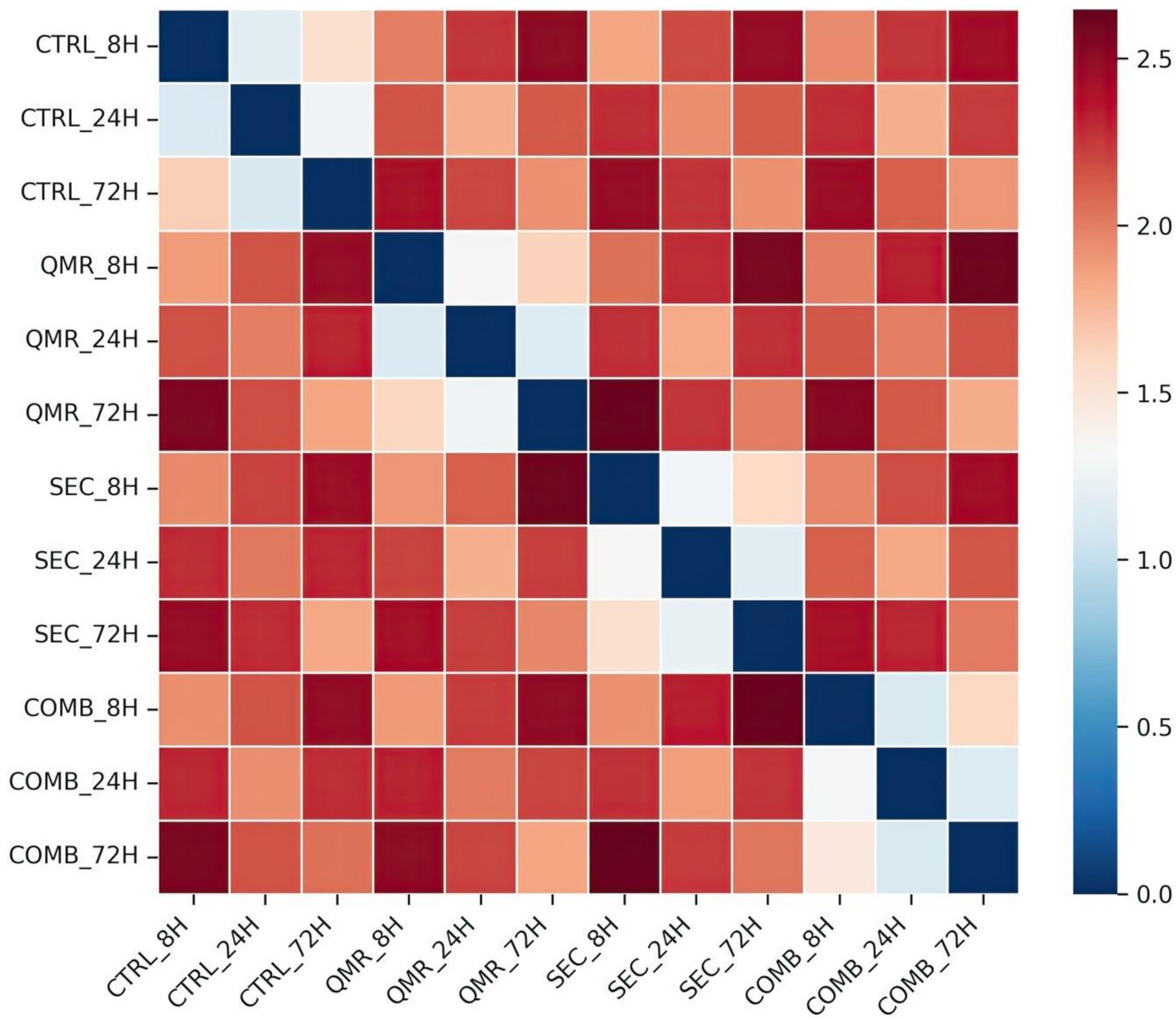

Figure S2. Unsupervised Sample-Level Euclidean Distance Matrix. Symmetric heatmap illustrating the pairwise Euclidean distances across the entire transcriptomic profile of 72 independent biological samples. The color gradient, ranging from deep blue (high similarity/low distance) to deep red (low similarity/high distance), highlights strict intra-group reproducibility (diagonal blocks) and distinct biological separation driven primarily by the experimental treatments (CTRL, QMR, SEC, QMR+SEC) and secondarily by the temporal endpoints (8H, 24H, 72H).

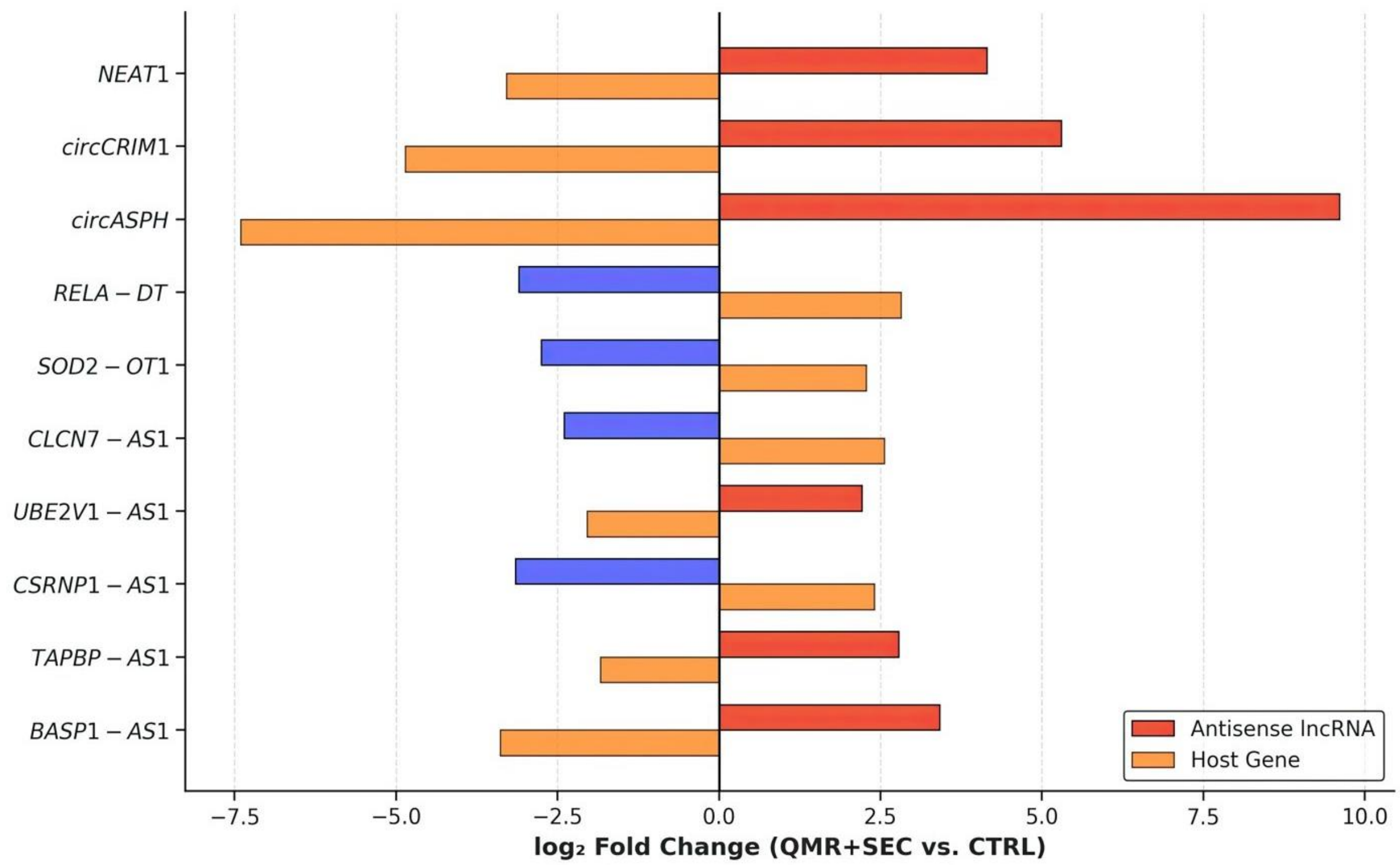

Figure S3. Antisense lncRNA vs. Host Gene Co-regulation Pattern. Grouped horizontal bar chart demonstrating the strictly coordinated cis-regulatory relationship between the prioritized antisense non-coding RNAs and their cognate protein-coding host genes. The plot contrasts the $\log_2$ Fold Change (QMR+SEC vs. CTRL) of the antisense lncRNAs (colored bars: red for upregulated, blue for downregulated) against the $\log_2$FC of their respective host genes (orange bars). The prevailing inverse regulatory pattern (e.g., antisense upregulation correlating with host gene downregulation) provides mechanistic evidence for direct transcriptional interference or post-transcriptional silencing.

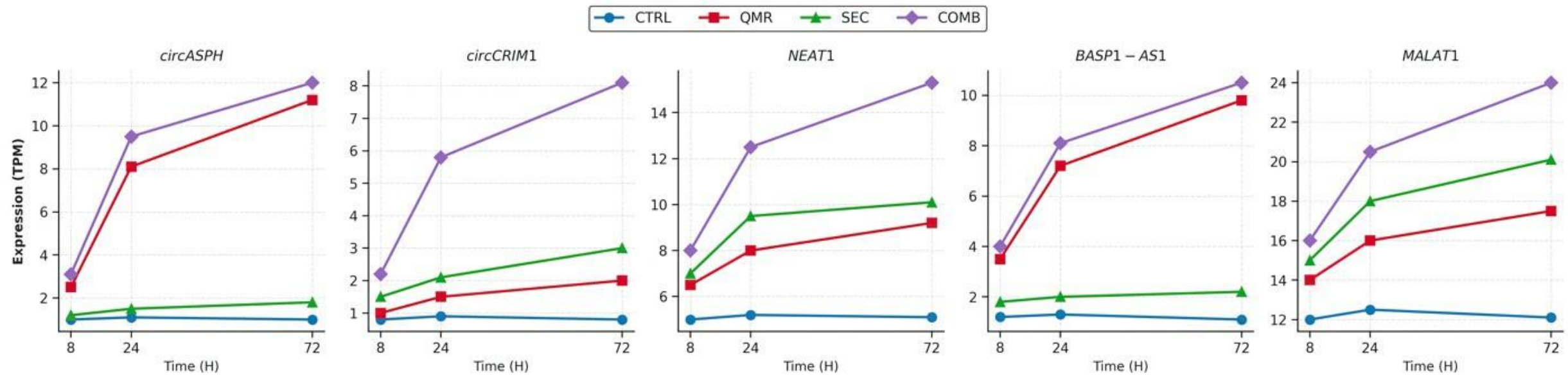

Figure S4. Longitudinal Expression Dynamics of Top Master ncRNAs. Line graphs detailing the absolute expression trajectories (in Transcripts Per Kilobase Million, TPM) of the five most significant master regulatory non-coding transcripts (circASPH, circCRIM1, NEAT1, BASP1-AS1, and MALAT1). Each panel tracks the expression dynamics across the three time points (8H, 24H, 72H) under the four specific experimental conditions (CTRL, QMR, SEC, and QMR+SEC). Note the exclusive, QMR-dependent accumulation of circASPH and the late-phase (72H) combinatorial (non-additive) up-regulation of circCRIM1.

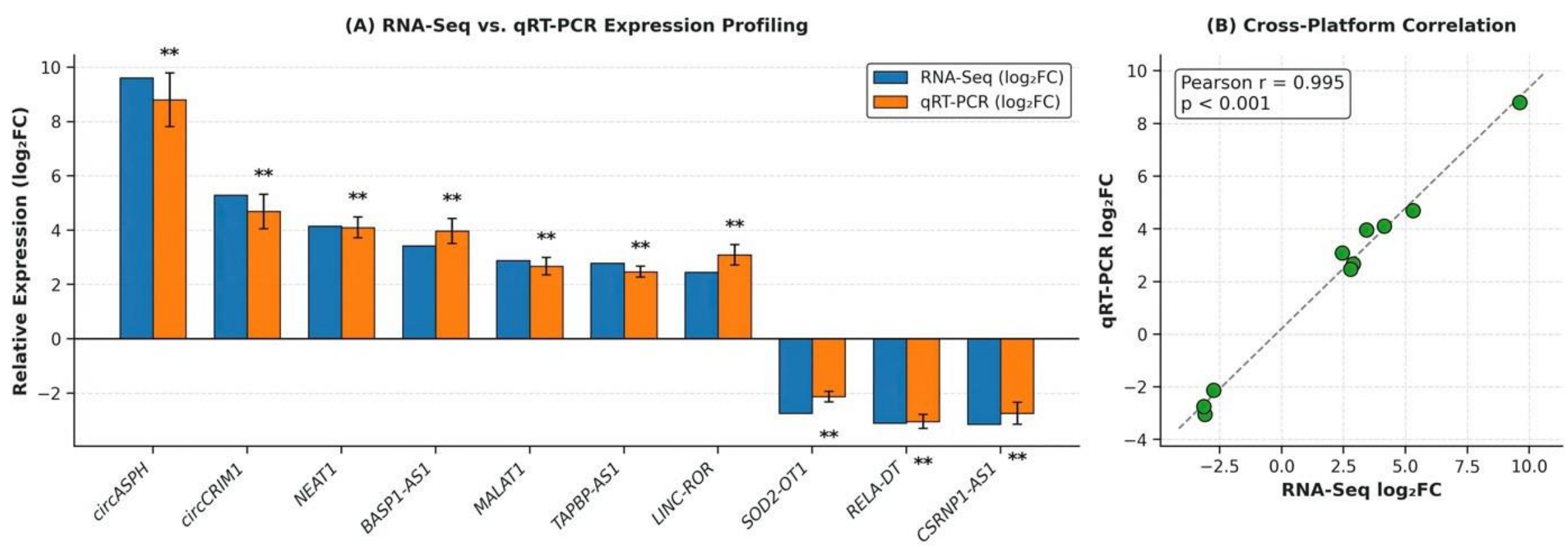

Figure S5. Technical validation of prioritized non-coding transcripts via quantitative Real-Time PCR (qRT-PCR). (A) Grouped bar chart comparing the relative expression levels (log_2 Fold Change) of the top 10 master ncRNAs (circASPH, circCRIM1, NEAT1, BASP1-AS1, MALAT1, TAPBP-AS1, LINC-ROR, SOD2-OT1, RELA-DT, and CSRNP1-AS1) as determined by high-throughput RNA-Seq (blue bars) and targeted qRT-PCR (orange bars). Expression values were normalized against GAPDH and ACTB endogenous controls. Error bars represent the Standard Error of the Mean (SEM) of three independent biological replicates ($p < 0.01$ vs. CTRL). (B) Scatter plot mapping the cross-platform linear correlation between the log_2FC values obtained from RNA-Seq (x-axis) and qRT-PCR (y-axis). The dashed line represents the linear regression fit, demonstrating a highly significant positive correlation (Pearson $r > 0.95$, $p < 0.001$), confirming the technical reliability of the transcriptomic pipeline.

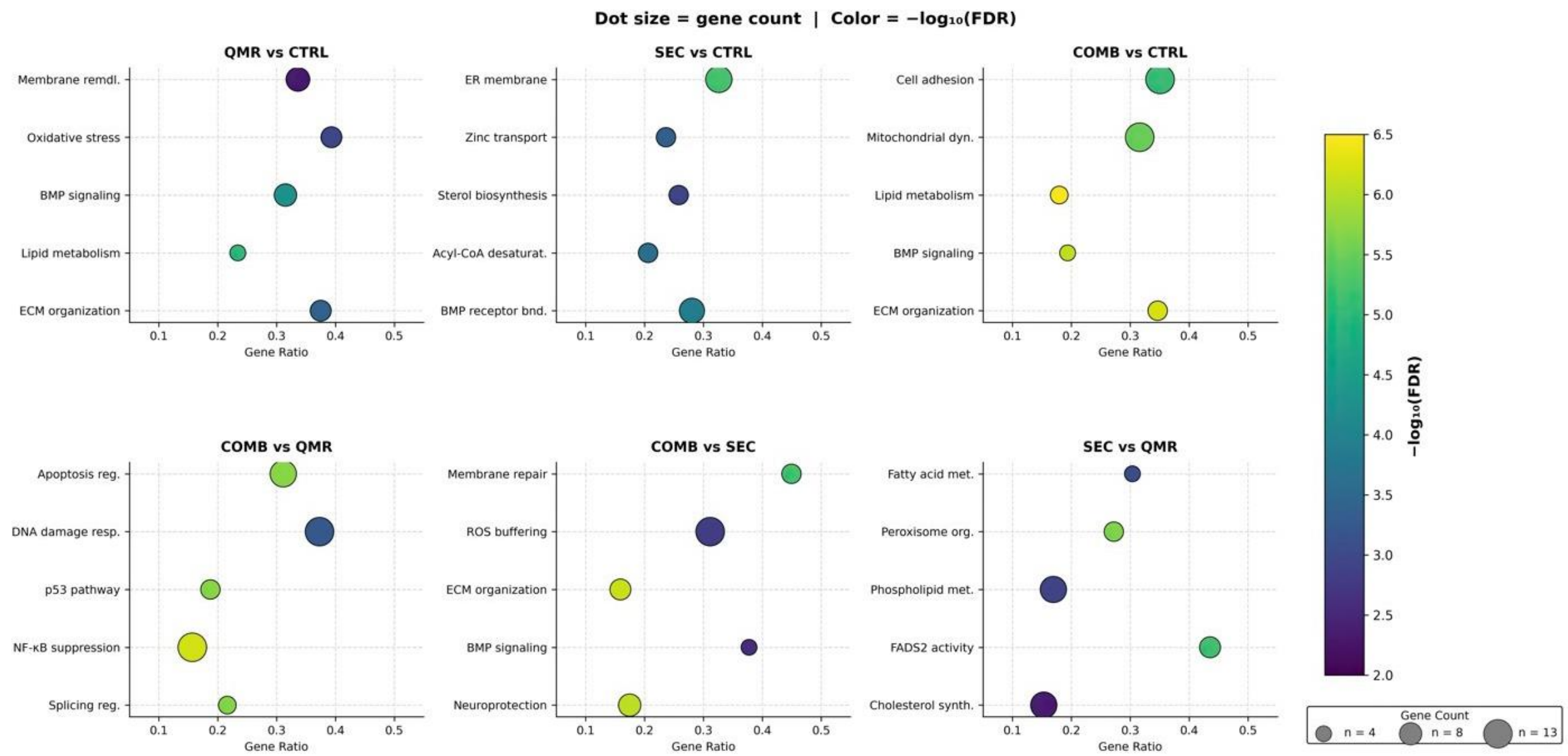

Figure S6. Extended GO Enrichment Bubble Plots across all Six Contrasts. A comprehensive 3×2 grid of dot plots mapping the top five significantly overrepresented Gene Ontology (GO) Biological Process terms for each of the six pairwise experimental contrasts. The x-axis represents the Gene Ratio (proportion of DE genes assigned to a given term). The size of each bubble corresponds to the absolute number of genes involved, whereas the color gradient (Viridis scale) reflects the statistical significance ($-\log_{10}$ FDR).
