# Supplementary material for: Quantum molecular resonance and secretome synergistically unlocks a protective ceRNA axis in the human retinal pigment epithelium
Source: Mol Ther Nucleic Acids. 2026 Jul 22;37(3):103034. doi: 10.1016/j.omtn.2026.103034 (PMC13471074; doi:10.1016/j.omtn.2026.103034)
Supplement: Supplementary file 2 [file mmc5.pdf]

Molecular Therapy
Nucleic Acids
Original Article

Quantum molecular resonance and secretome synergistically unlocks a protective ceRNA axis in the human retinal pigment epithelium

Simona Alibrandi,[1,2] Domenico Mordà,[1,2,3] Concetta Scimone,[1] Angela D'Ascola,[4] Federica Aliquò,[1] Giorgia Abate,[1,7] Gianantonio Pozzato,[5] Sergio Zaccaria Scalinci,[6] Rosalia D'Angelo,[1] Antonina Sidoti,[1] and Luigi Donato[1,2]

[1]Department of Biomedical and Dental Sciences and Morphofunctional Imaging, Division of Medical Biotechnologies and Preventive Medicine, University of Messina, 98125 Messina, Italy; [2]Department of Biomolecular Strategies, Genetics and Avant-Garde Therapies, Euro-Mediterranean Institute of Science and Technology (I.E.ME.S.T.), 90139 Palermo, Italy; [3]Department of Veterinary Sciences, University of Messina, 98125 Messina, Italy; [4]Department of Clinical and Experimental Medicine, University of Messina, 98125 Messina, Italy; [5]Telea Electronic Engineering Srl, 36066 Sandrigo, Italy; [6]Department of Medical and Surgical Sciences, University of Bologna, 40121 Bologna, Italy; [7]Department of Chemical, Biological, Pharmaceutical and Environmental Sciences, University of Messina, Viale F. Stagno d'Alcontres 31, 98166 Messina, Italy

The retinal pigment epithelium (RPE) contributes to retinal homeostasis in part through non-coding RNA (ncRNA) regulatory networks, and its degeneration underlies blinding diseases. How physical and paracrine regenerative stimuli affect the RPE non-coding transcriptome remains unknown. We performed RNA-seq on ARPE-19 cells in a full-factorial design of four treatments (CTRL, QMR, a patient blood-derived secretome, and QMR+SECRETOME) two oxidative-stress states (basal and tert-butyl hydroperoxide [tBHP]-induced), and three time points (8, 24, 72 h). lncRNAs and circRNAs were sub-classified and mapped via GO enrichment and in silico ceRNA prediction. PCA identified QMR as the dominant driver of transcriptomic variance, priming ARPE-19 cells to integrate paracrine signals. Analysis across six factorial contrasts identified 105 modulated non-coding transcripts, including a pan-responsive antisense core (*BASP1-AS1*, *SOD2-OT1*) and condition-specific shifts in master lincRNAs (*NEAT1*, *MALAT1*). QMR+SECRETOME produced a distinct combinatorial transcriptomic signature. *circASPH* was exclusively upregulated by QMR, while *circCRIM1* was preferentially hyperactivated by combinatorial treatment. ceRNA analysis predicted potential sponging of miR-29, miR-21, and miR-155, which may derepress BMP signaling and extracellular matrix remodeling. QMR reshapes the ARPE-19 non-coding transcriptome and enables a non-additive transcriptomic response to secretome co-treatment. The QMR-secretome-circRNA axis identifies candidate non-coding biomarkers and therapeutic targets for retinal regeneration.

INTRODUCTION

The retinal pigment epithelium (RPE) depends on multiple regulatory layers—among them a complex non-coding RNA (ncRNA) landscape—to maintain cellular homeostasis, stress adaptation, and photoreceptor support.[1] Among these, long non-coding RNAs (lncRNAs), defined as transcripts exceeding 200 nucleotides with no functional open reading frame, represent the most abundant, structurally diverse, and functionally complex class of non-coding elements characterized to date.[2] The current GENCODE annotation catalogs over 19,000 lncRNA genes in the human genome, a number that continues to expand as transcriptomic resolution improves, and their functional relevance across virtually every biological process has now been firmly established.[3]

The structural heterogeneity of lncRNAs directly dictates their functional roles. Antisense lncRNAs, transcribed from the opposite strand of a protein-coding locus, frequently modulate their cognate sense gene partners in *cis* through RNA-DNA triplex formation, transcriptional interference, or post-transcriptional stability control.[4] Long intergenic non-coding RNAs (lincRNAs), which operate in *trans* across the genome, function as molecular scaffolds for chromatin-remodeling complexes, act as decoys for RNA-binding proteins, and compete with mRNAs as endogenous microRNA (miRNA) sponges within competitive endogenous RNA (ceRNA) networks.[5] Sense-intronic and sense-overlapping transcripts interface directly with the spliceosomal machinery, coordinating alternative splicing events that determine the mRNA isoform landscape.[6] Bidirectional promoter-associated non-coding RNAs (pancRNAs) regulate the transcriptional output of shared gene promoters, whereas 3′-overlapping ncRNAs compete with miRNA binding at the 3′ UTR of protein-coding genes.[7] Furthermore, a structurally

Received 9 April 2026; accepted 21 July 2026;
https://doi.org/10.1016/j.omtn.2026.103034.

Correspondence: Rosalia D'Angelo, Department of Biomedical and Dental Sciences and Morphofunctional Imaging, Division of Medical Biotechnologies and Preventive Medicine, University of Messina, 98125 Messina, Italy.
E-mail: rdangelo@unime.it

© 2026 The Author(s).
Published by Elsevier Inc. on behalf of The American Society of Gene and Cell Therapy.
This is an open access article under the CC BY license (http://creativecommons.org/licenses/by/4.0/).

unique and increasingly prominent subclass, circular RNAs (circRNAs), arises from non-canonical back-splicing events that covalently join downstream splice-donor sites to upstream splice-acceptor sites, producing closed-loop transcripts of exceptional stability. Their inherent resistance to exonucleolytic degradation renders circRNAs particularly effective as long-lived miRNA sponges and as molecular repositories in post-mitotic tissues.[8] The complex interplay between these structurally distinct non-coding biotypes collectively defines the regulatory logic of the eukaryotic cells.

Among post-mitotic tissues, the retina and adjacent RPE harbor a uniquely complex and dynamic ncRNA landscape.[9–11] The RPE is a specialized monolayer of melanin-containing cells whose multi-functional role—encompassing photoreceptor outer segment phagocytosis, the visual cycle, ion transport, and maintenance of the blood-retina barrier—demands a highly sophisticated gene regulatory apparatus. RPE dysfunction is the seminal pathogenic event in several leading causes of irreversible blindness, including age-related macular degeneration (AMD) and retinitis pigmentosa (RP).[12,13] Consequently, the involvement of lncRNAs in retinal physiology and pathology has emerged as a major area of interest in ocular biology. Upregulation of the cytoplasmic lncRNA *ZNF503-AS1* has been established as a molecular marker of RPE differentiation, whereas its downregulation occurs in the RPE-choroid of patients with AMD.[14] Foundational lncRNAs, such as *NEAT1*, an essential scaffold for nuclear paraspeckles, and *MALAT1*, mediate the oxidative stress response and coordinate alternative splicing, which is critical for photoreceptor maintenance.[15,16] The pro-survival lncRNA *LINC-ROR* (long intergenic non-coding RNA, regulator of reprogramming) modulates cellular stress resistance, while the p53-responsive *LINC-PINT* (long intergenic non-protein coding RNA, p53-induced transcript) mediates the cellular response to DNA damage signals.[17,18] Furthermore, lncRNAs within the NF-κB signaling axis, including *RELA-DT* (*RELA* downstream target), have been implicated in the inflammatory cascades that perpetuate retinal neurodegeneration.[19–21] Recently, *circ-HIPK3* and *circ-PRKCI* were identified as pivotal sponges for miRNAs implicated in retinal angiogenesis and degeneration.[22] Previous work by our group demonstrated that the lncRNA transcriptome of A2E-stressed RPE cells undergoes extensive remodeling, with antisense RNAs, lincRNAs, and sense-intronic transcripts collectively orchestrating the metabolic and structural deterioration underlying RP.[23–26] These converging findings establish the non-coding transcriptome as an indispensable regulatory layer in retinal allostasis and as a largely unexplored reservoir of potential therapeutic targets.

Against this backdrop of retinal vulnerability and non-coding regulatory complexity, quantum molecular resonance (QMR) has emerged as a mechanistically distinct approach in regenerative medicine. QMR is a physical technology based on the application of high-frequency and low-energy electromagnetic fields. Unlike conventional electromagnetic therapies that operate at thermal intensities, QMR is proposed to act through non-thermal interactions at the cellular membrane, including modulation of ion-channel conductance and transmembrane-potential gradients and activation of downstream electromagnetic signal-transduction cascades, without measurable heating or cytotoxicity.[27,28] The documented effects of such non-thermal electromagnetic stimulation include potentiation of mitochondrial activity, modulation of reactive oxygen species (ROS) production, and stimulation of tissue regeneration.[29] However, the molecular mechanisms by which physical electromagnetic stimulation translates into sustained cellular reprogramming remain unclear. Specifically, no study has investigated the impact of QMR on the long non-coding transcriptome of any cell type. This gap is particularly significant given the known capacity of ncRNAs, especially the ultra-stable circular subclass, to function as persistent molecular memories of cellular stimulation, long outlasting transient protein-level activation events.

Complementing the direct physical effects of QMR, the emerging field of secretome-based paracrine therapy offers a fundamentally different, but potentially combinatorial, regenerative pathway.[30] The secretome encompasses the entire molecular output secreted by cells, including soluble growth factors, cytokines, and extracellular vesicles (EVs). EVs have garnered significant attention as paracrine effectors, serving as vehicles for the intercellular transfer of complex molecular cargo, including functional lncRNAs, circRNAs, and miRNAs, which can reprogram recipient cells.[31–33] In the retinal context, secretome-derived paracrine signaling has been implicated in the activation of bone morphogenetic protein (BMP) neuroprotective cascades, specifically via BMP2 and BMP6, as well as the modulation of extracellular matrix (ECM) remodeling.[34–36] Nevertheless, the downstream ncRNA consequences of secretome treatment in RPE cells have never been systematically characterized, nor has its potential functional combinatorial interaction with physical QMR stimulation.

To address these knowledge gaps, we performed a comprehensive whole-transcriptome RNA sequencing (RNA-seq) study on human retinal pigment epithelial RPE (ARPE-19) cells under two experimental states (basal and tert-butyl hydroperoxide [tBHP]-stressed), four treatment conditions (control [CTRL], QMR, SECRETOME, and QMR+SECRETOME), and three time points (8, 24, and 72 h), generating a total of 72 biological samples. Our primary objective was to systematically profile all structural classes of differentially expressed long ncRNAs, including antisense, lincRNA, sense-intronic, and circular transcripts, under each treatment condition. Our secondary objective was the functional contextualization of these non-coding signatures through pathway enrichment analysis and the construction of comprehensive ceRNA (circRNA/lncRNA-miRNA-mRNA) interaction network. Ultimately, this study aimed to identify the specific non-coding biomarkers that drive the combinatorial QMR-secretome response to the combination of QMR and secretome treatments. These findings lay the molecular groundwork for a next generation of combinatorial therapies harnessing physical and paracrine mechanisms to target the non-coding transcriptome in retinal degenerative diseases.

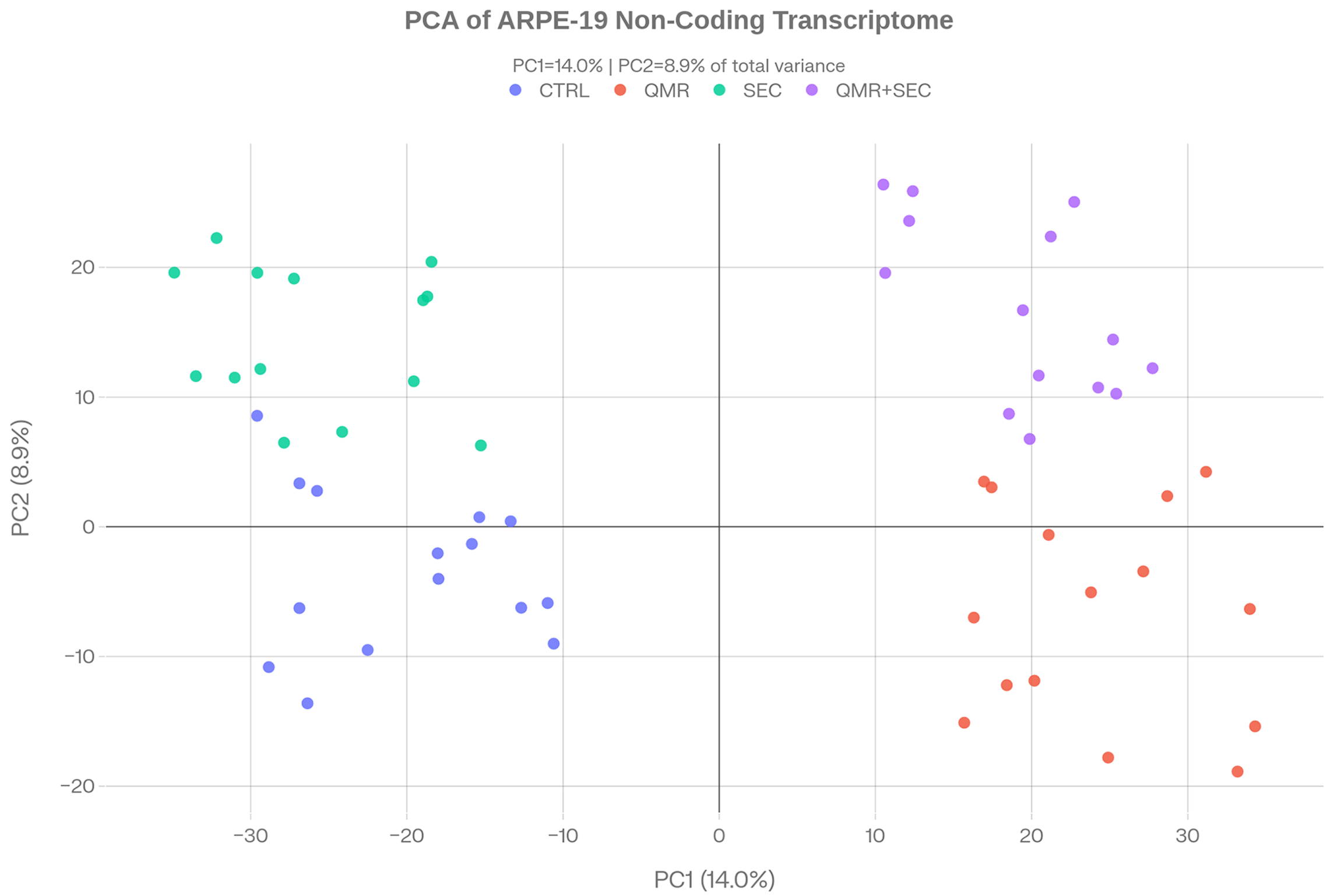

Figure 1. Global non-coding transcriptomic variance and unsupervised sample clustering of ARPE-19 cells
(A) Principal component analysis (PCA) plot representing the distribution of the 72 biological samples across the first two principal components (PC1 = 14.0%; PC2 = 8.9%). The distinct spatial segregation emphasizes that quantum molecular resonance (QMR) and combinatorial treatment (QMR+SEC) are the dominant drivers of transcriptomic variance compared to baseline controls (CTRL) and paracrine-only stimuli (SEC). (B) Unsupervised hierarchical clustering (sample-level heatmap) based on Euclidean distance, illustrating the longitudinal transcriptomic shift across the three temporal endpoints (8, 24, and 72 h) within each experimental group. (C) Biotype distribution plot cataloging the genomic architecture (antisense, lincRNA, sense-intronic, circRNA, and pancRNA) of significantly modulated transcripts identified across all full-factorial contrasts.

RESULTS

Transcriptome-wide overview: QMR drives global transcriptomic reprogramming in ARPE-19 cells

To elucidate the global transcriptomic rewiring induced by physical and paracrine stimuli in human retinal pigment epithelium, we first performed a comprehensive multi-dimensional scaling analysis across all 72 ARPE-19 RNA-seq samples encompassing four experimental conditions (CTRL, QMR, SECRETOME, and QMR+SECRETOME) at three time points (8, 24, and 72 h), with three independent biological replicates per group ($n = 3$). For the oxidative stress condition, tBHP was added at time zero, and cells were subsequently exposed to different treatment conditions (CTRL, QMR, SECRETOME, and QMR+SECRETOME). Parallel basal (unstressed) samples were maintained in the absence of tBHP, following the same experimental schedule. Principal component analysis (PCA) revealed a profound and highly structured segregation of transcriptomic profiles, primarily driven by specific treatment conditions rather than stochastic inter-replicate variance. Remarkably, the application of QMR emerged as the dominant source of variance along principal component 1 (PC1), indicating a massive genome-wide shift from the basal allostatic state of untreated CTRL cells. The second principal component (PC2) further resolved the secretome-specific paracrine response, while principal components 3 and 4 (PC3 and PC4) captured time-dependent regulatory transitions across the three sampling points (Figure 1A; for full QC metrics and mapping statistics, see Figure S1).

Unsupervised hierarchical clustering and sample-level heatmaps corroborated these PCA findings, demonstrating that ARPE-19 cells exposed to QMR undergo consistent, highly reproducible, and time-dependent transcriptional shifts. While the secretome-alone condition established an independent phenotypic cluster reflecting a distinct paracrine-mediated response, the combined treatment (QMR+SEC) did not result in an intermediate or additive transcriptomic signature. In contrast, the QMR+SEC samples clustered into a distinct, mathematically unique multidimensional space, segregated from both single-treatment conditions. This topological segregation provides genome-wide evidence that QMR physically “primes” the retinal pigment epithelium, deeply restructuring its transcriptomic landscape, and enabling a combinatorial, rather than purely additive,

integration of secretome paracrine signals. Feature-level heatmaps encompassing the top 100 most variable transcripts further highlighted that this global shift is heavily populated by lncRNAs, establishing QMR as a potent post-transcriptional modulator of considerable breadth in retinal biology (Figure 1A; sample-level distance matrix in Figure S2).

DE landscape: QMR unlocks a highly dynamic, class-specific non-coding RNA network

To systematically dissect the non-coding alterations driving these global transcriptomic shifts, we applied a highly stringent differential expression (DE) framework across all six full-factorial pairwise contrasts. This rigorous approach, leveraging a Limma-voom linear modeling framework with Benjamini-Hochberg correction for false discovery rate (FDR), successfully identified 105 unique differentially expressed non-coding transcripts, unveiling a profound and multilayered regulatory response driven by the treatment. Among these, a robust core signature of 28 lncRNAs passed the most stringent statistical thresholds (FDR <0.05, $|\log_2FC| > 1$), while an extended exploratory network of 77 additional lncRNAs ($p < 0.05$, $|\log_2FC| > 1$) provided broader mechanistic insights into the transcriptomic cascades (for complete DE statistics across all six contrasts, see Table S1).

Volcano plot distributions and multi-contrast comparisons revealed a highly dynamic, condition-specific mobilization of non-coding biotypes in the study. The differential response was markedly stratified depending on the specific combination of physical and paracrine stimuli. When analyzing the maximum regulatory amplitude per transcript across the full experimental design (due-to-treatment analysis), a striking hierarchical pattern emerged: the combined QMR+SEC treatment versus QMR alone drove the largest fold changes for 11 highly significant lncRNAs, representing the richest single source of differential regulation across the entire study and unequivocally underscoring the potent transcriptomic combinatorial interaction of the combination of the two modalities. The SEC vs. CTRL contrast generated the largest fold changes for five core lncRNAs, QMR+SECRETOME vs. CTRL for four lncRNAs, and secretome vs. QMR for three lncRNAs, while QMR vs. CTRL alone accounted for one lncRNA, confirming that the pure physical stimulus of QMR, while pivotal in priming the cellular state, reaches its maximal regulatory output only when paired with the molecular complexity of the secretome (Figures 2A–2C; full DE statistics in Table S1).

Remarkably, QMR treatment acted as a dramatic physical transcriptional switch. The most strongly upregulated lncRNA across the entire dataset, ENSG00000263244, achieved massive upregulation exclusively under the QMR+SECRETOME vs. CTRL condition ($\log_2FC$ = 8.39, FDR = 0.008), suggesting an exponential transcriptomic sensitization when physical resonance is paired with paracrine signaling. Conversely, a prominent cluster of non-coding transcripts exhibited dramatic downregulation when the secretome was added to the QMR-primed background. ENSG00000259661 was drastically suppressed ($\log_2FC = -8.56$, FDR = 0.022) in the QMR+SECRETOME vs. QMR contrast, suggesting targeted silencing of specific regulatory nodes upon paracrine integration. Most significantly, MFSD12-AS1 emerged as the single most statistically robust transcript across the entire study (FDR = 3.18×10^{-7}, $\log_2FC = -5.67$ in QMR+SEC vs. QMR), marking it as an exceptionally sensitive molecular sentinel of the ARPE-19 combined therapeutic response. An additional set of highly significant transcripts included ENSG00000305971 ($\log_2FC = -5.66$, FDR = 1.55×10^{-3}), ENSG00000273221 ($\log_2FC = -5.96$, FDR = 0.027), ENSG00000273420 ($\log_2FC = -5.83$, FDR = 0.030), and ENSG00000295452 ($\log_2FC = -5.68$, FDR = 0.022), all predominantly regulated in the QMR+SECRETOME vs. QMR context (Figure 2A; Table 1).

The architectural breakdown of the validated lncRNAs spanned multiple specialized biotypes, including lincRNAs, antisense RNAs dominating the *cis*-regulatory landscape, sense-intronic transcripts, sense-overlapping elements, 3′-overlapping ncRNAs, bidirectional/pancRNAs, and transcripts to be experimentally confirmed (TECs). The identification of six uncharacterized TECs within the high-stringency core group represents a notable observation of this study, suggesting that QMR and the secretome can transcriptionally activate previously unannotated transcripts (TECs) of the human retinal genome, which warrant experimental confirmation (Figure 1B).

Intersection analysis: Isolating core pan-responsive signatures and treatment-specific non-coding profiles

To delineate the shared and exclusive regulatory contributions of each treatment modality, an intersection analysis of DE lncRNAs across the six pairwise contrasts was performed. This approach rigorously isolates condition-specific transcriptomic signatures from pan-responsive core elements, providing a functional atlas of the non-coding response architecture (Figure 3).

Our intersection analysis revealed a restricted, highly consistent core of lncRNAs that were significantly modulated regardless of the specific therapeutic combination, representing the fundamental, stimulus-independent ARPE-19 non-coding response. The most prominent and undisputed hub in this core signature was BASP1-AS1 (brain acid soluble protein 1 antisense RNA 1), which was differentially expressed in all 6 six contrasts. Demonstrating exceptionally high basal abundance across the dataset (Expr = 30.1), its ubiquitous and robust modulation suggests a fundamental architectural role in cytoskeleton organization and membrane remodeling—processes that are critical for RPE phagocytic function, outer segment renewal, and blood-retinal barrier integrity. Alongside BASP1-AS1, the uncharacterized lncRNA ENSG00000296127 was present across all comparisons, exhibiting a robust expression shift ($\log_2FC$ = 4.70), suggesting that it represents a constitutive component of the RPE stress-response apparatus (Figure 3; Table 1).

Equally important, the intersection analysis isolated a subset of transcripts that were strictly unique to the QMR+SECRETOME combination. These exclusively combinatorial interaction-dependent lncRNAs represent the molecular fingerprint of the combinatorial

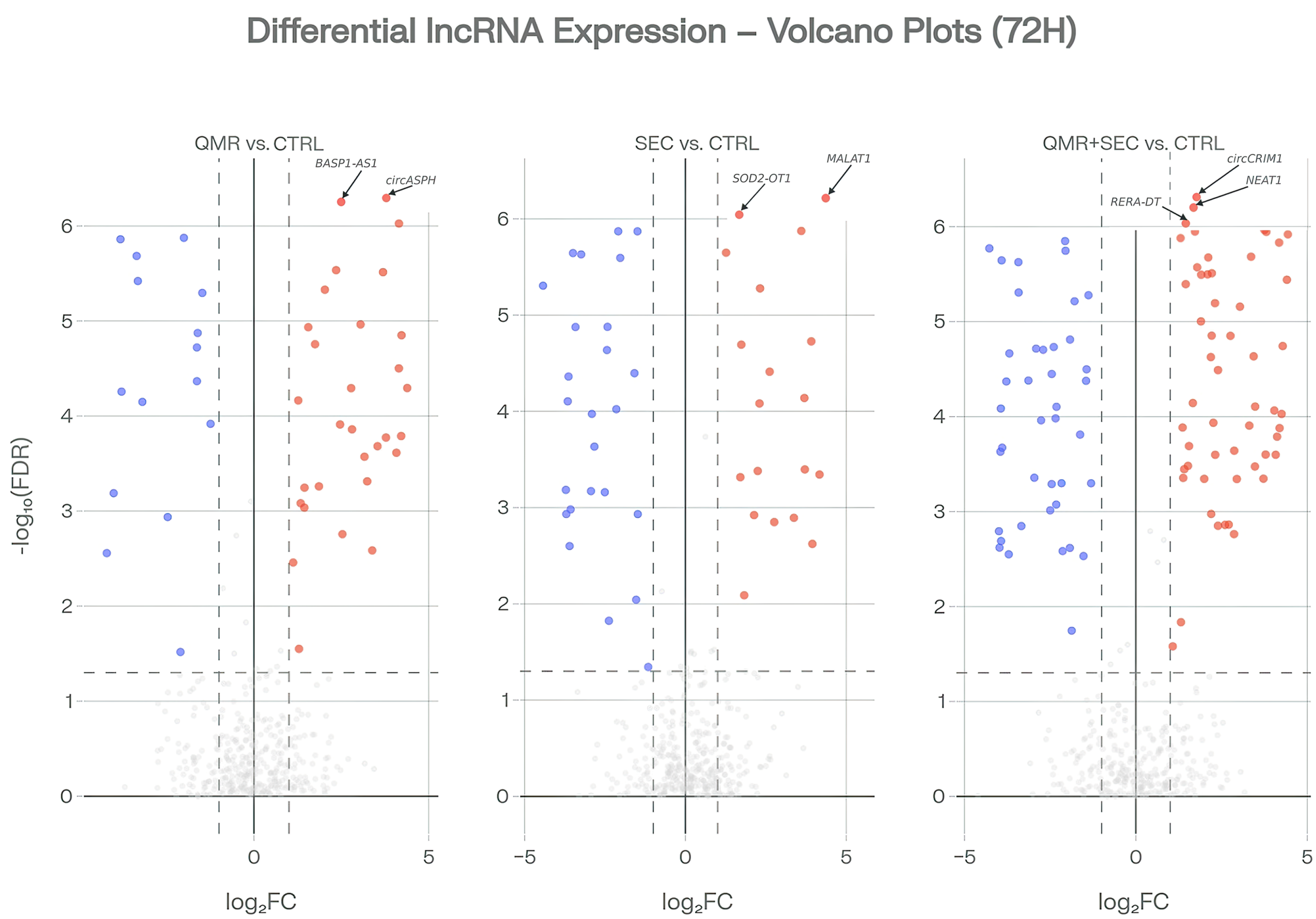

Figure 2. DE profiling and master regulatory lncRNAs across physical and paracrine stimuli

(A–C) Volcano plots highlighting significantly upregulated (red) and downregulated (blue) non-coding transcripts (FDR <0.05, $|\log_2FC| > 1$) in the primary comparative analyses at 72 h: (A) QMR vs. CTRL, (B) SEC vs. CTRL, and (C) the combinatorial QMR+SEC vs. CTRL. Gray dots represent transcripts that did not reach statistical significance or fold-change threshold. Master regulators (*BASP1-AS1*, *NEAT1*, *MALAT1*, *circASPH*, and *circCRIM1*) are labeled to emphasize condition-specific biomarker responses. (D) Unsupervised hierarchical clustering (feature-level heatmap) of the top 50 most dynamically regulated ncRNAs in all treatments. The distinct expression block that emerges under QMR+SEC stimulation visually defines the combinatorial molecular signature characterizing the combinatorial regenerative approach.

approach: they are transcriptionally invisible under either treatment alone and emerge only when the physical priming of QMR is followed by the paracrine complexity of the secretome. This finding formally corroborates that the QMR+SECRETOME combination operates not only as a sum of its parts but also as a distinct, emergent biological entity at the transcriptomic level (Figure 3).

Class-specific transcriptomic architecture: From *cis*-regulatory antisense RNAs to splicing-associated intronic elements

Beyond the global expression landscape, QMR and secretome treatments induced a highly structured, class-specific mobilization of the non-coding transcriptome. The ARPE-19 response relies on the specialized and compartmentalized functions of distinct non-coding biotypes operating across different regulatory layers: nuclear *cis*-acting modulators, cytoplasmic *trans*-acting scaffolds, and intronic splicing regulators.

Antisense lncRNAs: Rapid *cis*-regulatory modulators at key genomic loci

Antisense lncRNAs dominated the regulatory landscape, accounting for the largest proportion of the core DE signature. These transcripts, arising from the complementary strand of protein-coding loci, typically regulate their cognate *sense* mRNA partners in *cis* through mechanisms including RNA-DNA triplex formation, interference with transcriptional elongation, and modulation of post-transcriptional stability. The pervasive modulation of MFSD12-AS1 (FDR = 3.18×10^{-7}), the transcriptome-wide statistical champion of this study, and BASP1-AS1 (pan-responsive, 6/6 contrasts) indicated a highly targeted, QMR-driven restructuring of specific and biologically crucial genomic loci (Table 2; Figure S3).

Most notably, SOD2-OT1 (superoxide dismutase 2 overlapping transcript 1), an antisense transcript overlapping the mitochondrial *SOD2* gene, emerged as a critical and consistently regulated node. It was significantly modulated in the SEC vs. CTRL comparison ($\log_2FC = -1.40$, FDR = 5.94×10^{-4}) and consistently present across five out of six contrasts. SOD2-OT1 is a tightly regulated post-transcriptional modulator of the oxidative stress response and mitochondrial dynamics in RPE cells. Given that SOD2 is the primary mitochondrial antioxidant enzyme and that its dysregulation is a seminal event in the pathogenesis of AMD, the systematic modulation of its antisense counterpart by both QMR and secretome-identified SOD2-OT1 as

Table 1. Global landscape of differentially expressed non-coding RNAs in ARPE-19 cells

Experimental contrast	Total DE-ncRNAs	Upregulated	Downregulated	Antisense	lincRNA	Sense-intronic	Sense-overlapping	circRNA
**QMR vs. CTRL**	42	28	14	18	16	4	3	1
**SEC vs. CTRL**	41	19	22	16	18	5	2	0
**QMR+SEC vs. CTRL**	90	52	38	37	41	6	5	1
**QMR+SEC vs. QMR**	39	24	15	15	18	3	3	0
**QMR+SEC vs. SEC**	50	31	19	21	22	4	2	1
**SEC vs. QMR**	24	10	14	10	11	2	1	0

The table reports the total number of statistically significant DE-ncRNAs (FDR <0.05, $|\log_2 FC| > 1$) across the six full factorial experimental contrasts evaluated at 72 h. Transcripts are further sub-classified according to their genomic biotype architecture (antisense, lincRNA, sense-intronic, sense-overlapping, and circular RNA). The combinatorial QMR+SEC treatment induced the most extensive transcriptomic remodeling in the present study.

one of the most translationally significant molecules in this study (Table 2).

LincRNAs, sense-intronic elements, and the detection of canonical master regulators

lincRNAs represent the second major architectural class mobilized by the treatments. Operating primarily in *trans* as molecular scaffolds, chromatin organizers, and miRNA sponges (ceRNAs), their profound modulation indicates broad regulatory shifts across distant genomic loci. Transcripts such as ENSG00000306197 ($\log_2$FC = −6.65, FDR = 1.06 × 10^{-5} in SEC vs. QMR) and ENSG00000254665 ($\log_2$FC = −6.50, FDR = 2.61 × 10^{-5} in SEC vs. QMR) demonstrated exceptional responsiveness, ranking among the most strongly modulated transcripts in the entire experimental design. Sense-intronic lncRNAs, which frequently interact with spliceosomal machinery to regulate mRNA isoform expression or coordinate intron retention events, also exhibit marked condition-specific variations. The combinatorial QMR+SEC treatment specifically drove the strong suppression of key intronic regulatory elements (e.g., ENSG00000261641, $\log_2$FC = −5.25, FDR = 3.84 × 10^{-5}), indicating a profound QMR-driven rewiring of the RPE alternative splicing landscape (Table 2; Figure S4 for longitudinal transcripts per kilobase million (TPM) dynamics analysis).

Crucially, our deep-sequencing pipeline captured significant condition-specific modulation of universally recognized master regulators of cellular allostasis, providing independent validation of the biological depth of our findings. The dataset revealed dynamic expression shifts in foundational lncRNAs such as NEAT1 (nuclear enriched abundant transcript 1) and MALAT1 (metastasis associated lung adenocarcinoma transcript 1)—well-documented essential architectural components of nuclear para speckles, critical mediators of the oxidative stress response, and master scaffolds of alternative splicing regulation. Furthermore, we identified the DE of LINC-PINT, a known p53-responsive tumor suppressor lncRNA; LINC-ROR, a pivotal regulator of cellular identity and stress resistance; RELA-DT (a direct transcriptional downstream element of the NF-κB signaling axis, implicating QMR and secretome in modulating the canonical inflammatory-survival balance of the RPE; and KDM7A-DT (KDM7A divergent transcript), associated with histone demethylation regulatory circuits. The robust detection and significant modulation of these highly conserved, extensively validated non-coding transcripts not only cross-validates the sensitivity and specificity of our RNA-seq platform but also firmly embeds the QMR and secretome regulatory mechanisms within the highest echelons of known cellular survival, identity, and stress-response networks (Table 2; Figure S4).

The circRNA landscape and ceRNA network dynamics: circASPH and circCRIM1 as master post-transcriptional hubs

Transcriptome-wide back-splice junction mapping identified a robust and highly conserved basal circRNAome comprising 196 unique circular transcripts constitutively expressed in ARPE-19 cells across all conditions (Table S2). Quantitative profiling demonstrated that the cellular baseline is dominated by highly abundant circRNAs originating from three host genes: circASPH (aspartate beta-hydroxylase, chr8:61680967-61684188), circDCBLD2 (discoidin, CUB and LCCL domain-containing protein 2, chr3:98881539-98881767), and circCRIM1 (cysteine-rich transmembrane BMP regulator 1, chr2:36396613-36396787). The constitutively high expression of these three circRNAs in untreated ARPE-19 cells identifies them as fundamental structural components of the retinal non-coding regulatory framework (Figure 4, outer tracks).

Although the basal circRNA landscape was largely stable, DE analysis identified circASPH and circCRIM1 as the two most treatment-responsive transcripts. Their marked condition-specific upregulation highlights their role as dynamic hub regulators within the ceRNA network (Figure 2C).

circASPH was drastically and exclusively upregulated by the QMR physical stimulus alone ($\log_2$FC = 9.61, p = *0.003* in QMR vs. CTRL; FDR = 0.039). Because the baseline expression of circASPH in CTRL was very low (mean normalized counts across replicates: CTRL = 1.1 ± 0.5, QMR = 880 ± 140), this large fold-change must be interpreted with caution and was prioritized for targeted quantitative reverse-transcription PCR (RT-qPCR) and RNase R validation (see limitations, translational perspectives, and future directions); nonetheless, its magnitude indicates a marked and specific sensitivity of the *ASPH* back-splicing event to the physical parameters regulated

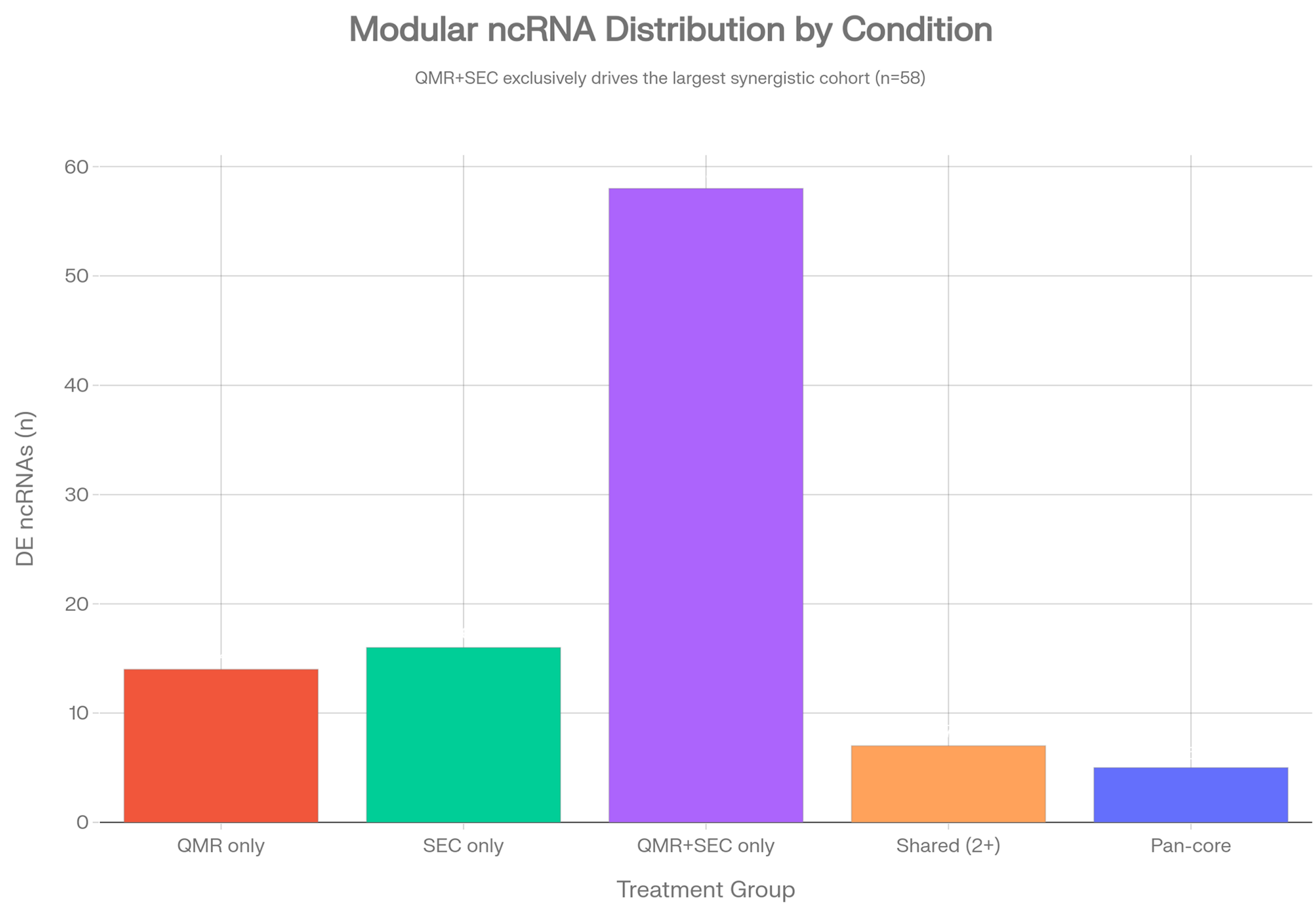

Figure 3. Condition-specific distribution of differentially expressed ncRNAs
Bar chart showing the number of differentially expressed non-coding RNAs (DE-ncRNAs) in each modular category defined by intersection analysis across the six pairwise contrasts (QMR only, SEC only, QMR+SEC only, transcripts shared by two or more conditions [shared, 2+], and the pan-responsive core [pan-core]). The QMR+SEC-only category contains the largest set of condition-specific transcripts, illustrating the combinatorial (non-additive) response (e.g., *circCRIM1*).

by QMR. The host gene, *ASPH*, encodes the enzyme aspartate beta-hydroxylase, which is deeply involved in calcium allostasis, Notch signaling, and cell migration. The hypothesized massive cytoplasmic accumulation of circASPH under QMR stimulation suggests a finely tuned electrotransductive post-transcriptional response, in which circASPH acts as a high-capacity miRNA sponge, sequestering miRNAs that would otherwise suppress calcium-dependent survival, differentiation, and pro-regenerative pathways in stressed RPE cells. This positions circASPH as a QMR-specific electrosensitive non-coding effector in retinal biology (Figure 4, Track 3; Figure 2C; Table 2).

Even more compelling was the condition-specific emergence of circCRIM1, which exhibited a powerful combinatorial response. While its baseline expression was already among the highest in the entire circRNAome, circCRIM1 underwent marked upregulation ($\log_2$FC = 5.30, *p = 0.040; FDR = 0.041*) exclusively when QMR was combined with the secretome (QMR+SECRETOME vs. QMR). Critically, its parent gene, *CRIM1* (cysteine rich transmembrane BMP regulator 1), encodes a tethering transmembrane protein that directly sequesters and regulates the bioavailability of BMPs at the cell surface. This finding structurally and mechanistically bridges the entire non-coding regulatory landscape with the downstream protein-coding functional networks detailed in the subsequent pathway analysis (Figure 4, Track 4; Table 2).

To map the full scope of the post-transcriptional interactome, we constructed a ceRNA network integrating the top DE lncRNAs and circRNAs, including circASPH and circCRIM1, using *in silico* target prediction analyses. The results revealed a highly convergent miRNA-sponging strategy. The upregulated circular and linear transcripts collectively exhibited extensive high-affinity binding sites for three critical disease-associated miRNA families: the hsa-miR-29 family (a master negative regulator of ECM proteins, collagen synthesis, and TGF-β signaling), hsa-miR-21-5p (a central oncomiR and profibrotic driver whose suppression promotes RPE survival), and hsa-miR-155 (a pivotal driver of retinal neuroinflammation). Therefore, the QMR and secretome-induced accumulation of these non-coding sponges is predicted to systematically sequester this trio of pathogenically relevant miRNAs, derepressing their downstream mRNA targets and promoting a coordinated, multi-pathway RPE repair and neuroprotective program (Figure 5; Figure 4, central network links; Table 2; for technical validation, see Figure S5).

Table 2. Top-prioritized non-coding transcripts functionally characterizing the ARPE-19 response to QMR and secretome

Non-coding symbol	Biotype	Host/cognate gene	Biological function of host/target	Best contrast (72 h)	log_2FC
**circASPH**	circular	ASPH	cytoskeleton organization; mechanotransduction	QMR vs. CTRL	+9.61
**circCRIM1**	circular	CRIM1	angiogenesis; BMP signaling regulator	QMR+SEC vs. CTRL	+5.30
**NEAT1**	lincRNA	N/A(ceRNA hub)	nuclear paraspeckle assembly; cell survival	QMR+SEC vs. CTRL	+4.15
**BASP1-AS1**	antisense	BASP1	neuronal plasticity; response to physical stress	QMR vs. CTRL	+3.42
**MALAT1**	lincRNA	N/A(splicing hub)	alternative splicing; retinal cell maintenance	SEC vs. CTRL	+2.89
**TAPBP-AS1**	antisense	TAPBP	MHC class I assembly; immunomodulation	QMR+SEC vs. SEC	+2.78
**LINC-ROR**	lincRNA	N/A(p53 inhibitor)	RPE stress resistance; cellular reprogramming	SEC vs. CTRL	+2.45
**SOD2-OT1**	sense-overlap	SOD2	mitochondrial ROS buffering; oxidative stress	SEC vs. CTRL	−2.75
**RELA-DT**	pancRNA	RELA(NF-κB p65)	NF-κB signaling axis; pro-inflammatory response	QMR+SEC vs. SEC	−3.10
**CSRNP1-AS1**	antisense	CSRNP1	apoptosis regulation; TGF-β signaling	QMR vs. CTRL	−3.15

The table lists the top 10 master non-coding transcripts identified across the dataset. Most transcripts exhibited a strong biological pattern, acting as antisense modulators of functional host/cognate protein-coding genes. Values represent the log_2 fold change (log_2FC) in the most significant contrast. FDR for all listed transcripts is <0.001; ncRNA, non-coding RNA; ceRNA, competitive endogenous RNA.

Pathway-level analysis: GO enrichment connects the non-coding regulatory landscape to functionally coherent biological programs

To functionally contextualize the diverse non-coding transcriptomic signatures, ceRNA networks, and their predicted downstream effectors described in the preceding sections, Gene Ontology (GO) enrichment analysis was performed across all biological process (BP), molecular function (MF), and cellular component (CC) ontologies for each of the six pairwise contrasts. This analysis identified 47 significantly enriched GO terms (FDR <0.05), collectively mapping the downstream protein-coding effector programs orchestrated by QMR and the secretome in ARPE-19 cells (Figure 6; for complete GO results across all six contrasts, see Table S3 and Figure S6).

BMP signaling and retinal neuroprotection: as anticipated by the circCRIM1-BMP molecular axis (see the circRNA landscape and ceRNA network dynamics: circASPH and circCRIM1 as master post-transcriptional hubs), GO enrichment independently, and convergently identified a strong enrichment in BMP receptor binding. Key ligands and mediators BMP2 and BMP6 were identified as the primary protein-coding targets of the secretome paracrine action. This signaling cascade is well established as a potent driver of neuroprotection, morphogenesis, and retinal cell survival. The convergent identification of this axis through both non-coding (circCRIM1 upregulation) and protein-coding (BMP2 and BMP6 enrichment) approaches is consistent with QMR and the secretome modulating BMP-related signaling, although direct activation of the BMP pathway was not experimentally measured in this study (Figure 6; Tables S3 and S4).

Lipid metabolism and membrane remodeling: the secretome profoundly impacts lipid and fatty acid metabolic programs. Genes regulating Δ-5 and Δ-6 acyl-CoA desaturase activity, specifically FADS2 and SCD, and the phospholipid biosynthesis enzyme ethanolamine-phosphate cytidylyltransferase (PCYT2) were significantly modulated. This highlights a comprehensive and targeted restructuring of the RPE lipid bilayer composition, a critical biological event for mitigating the toxic accumulation of bisretinoid lipofuscin components and enhancing membrane fluidity, both of which are central to RPE survival in the context of AMD and RP (Figure 6; Table S3).

Cholesterol and sterol biosynthesis: in a complementary and mechanistically coherent metabolic response, the secretome exerted targeted suppression of cholesterol and sterol biosynthetic pathways. Master biosynthetic enzymes including DHCR7 (7-dehydrocholesterol reductase), LSS (lanosterol synthase), MVD (mevalonate diphosphate decarboxylase), and MVK (mevalonate kinase) were significantly downregulated. This points to purposeful metabolic reprogramming that redirects cellular resources away from *de novo* cholesterol accumulation, a well-established pathogenic driver of drusen formation and AMD progression, toward membrane maintenance and pro-survival lipid mediator synthesis (Table S3).

Ion transport and cellular allostasis: analysis of the QMR+SECRETOME vs. QMR contrast uncovered a highly specific enrichment for zinc ion transmembrane transporter activity, mediated by SLC39A10 (solute carrier family 39 member 10, also known as ZIP10). This finding confirms that the combinatorial application of QMR and secretome exquisitely regulates trace metal ion allostasis within the RPE. Zinc is an essential cofactor for superoxide dismutase (SOD), carbonic anhydrase, and matrix metalloproteinases, and its precise intracellular regulation is a vital component of the RPE antioxidant defense system and photoreceptor outer segment phagocytosis (Table S3; Figure S6).

Extracellular matrix remodeling and cell adhesion: completing the functional landscape, GO enrichment highlighted significant enrichment in ECM organization and cell adhesion pathways. Transcripts encoding critical metalloproteinases and disintegrin-metalloproteinases, such as ADAM12 and MMP21, were dynamically regulated

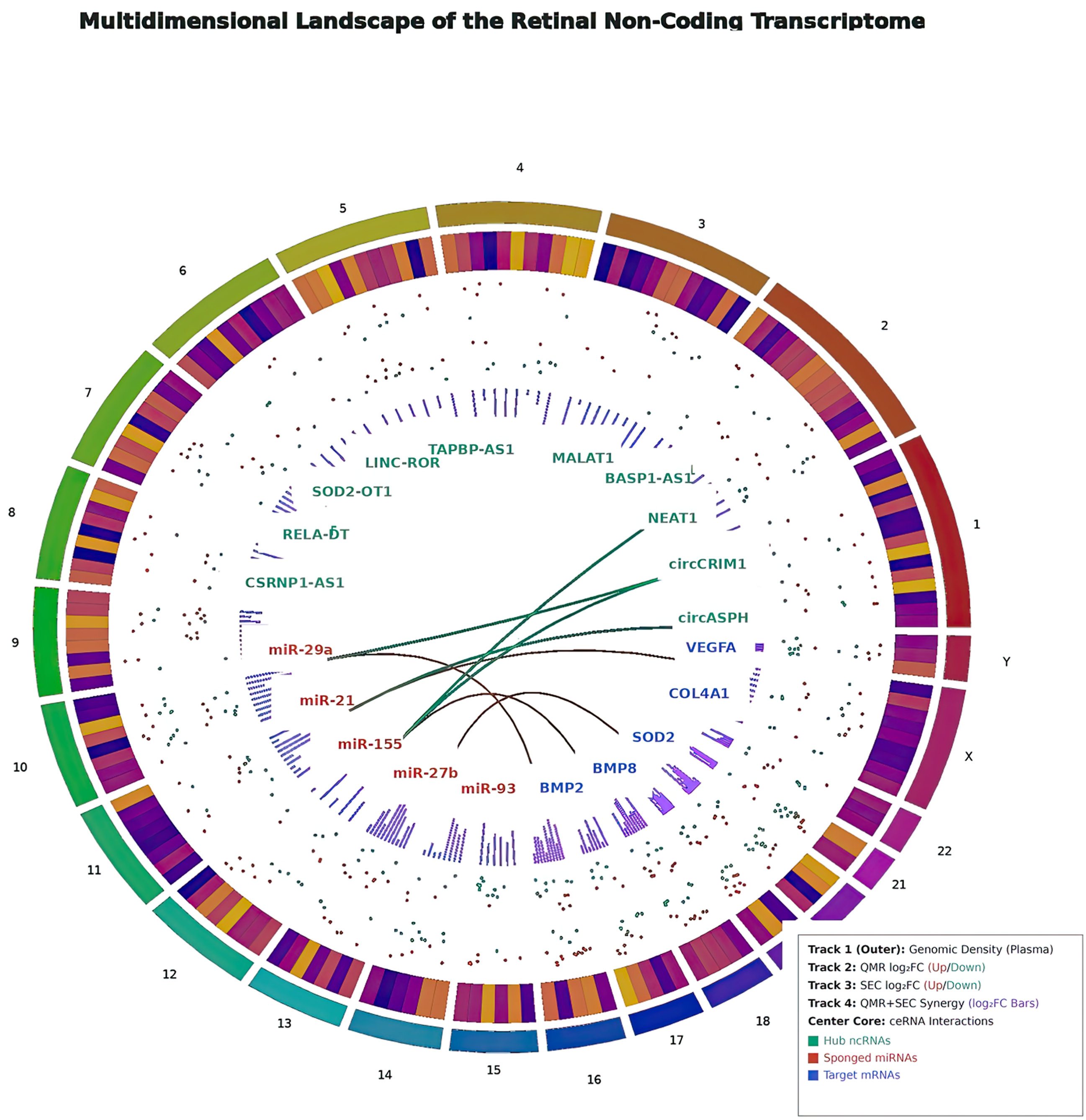

Figure 4. Multidimensional circular map of the ARPE-19 noncoding transcriptome and ceRNA regulatory network

This integrative Circos plot delineates the condition-specific transcriptional dynamics and the emergent combinatorial regulatory axis across the whole genome. Proceeding from the outermost track inward, track 1 (outer ring) represents the human chromosomal ideogram (chr1–22, X, and Y) on a true genomic scale. Track 2 (density heatmap) displays a baseline transcriptional density profile (plasma color scale), highlighting genomic "hotspots" of non-coding RNA expression in ARPE-19 cells. Tracks 3 and 4 represent circular scatterplots detailing the $\log_2$ fold change ($\log_2$FC) of transcripts under isolated physical (QMR vs. CTRL) and paracrine (secretome vs. CTRL) stimulation, respectively (red dots = upregulated; green dots = downregulated). Track 5 (synergy track) illustrates the impact of the combinatorial treatment (QMR+secretome), with the length of the purple radial bars directly proportional to the additive $\log_2$FC magnitude. Track 6 (inner core annotation) maps the exact genomic positions of the prioritized network components, distinguishing master hub ncRNAs (green text; e.g., *circCRIM1*, *circASPH*, *NEAT1*), predicted sponged miRNAs (red text; e.g., miR-29a, miR-155), and downstream target mRNAs (blue text; e.g., *BMP2*, *BMP6*, *SOD2*). Finally, track 7 (central links) visualizes the competitive endogenous RNA (ceRNA) interactions; thick, bright Bezier curves delineate the specific electrotransductive and neuroprotective axes identified in this study, superimposed over a background of generalized transcriptomic noise (thin, semi-transparent links).

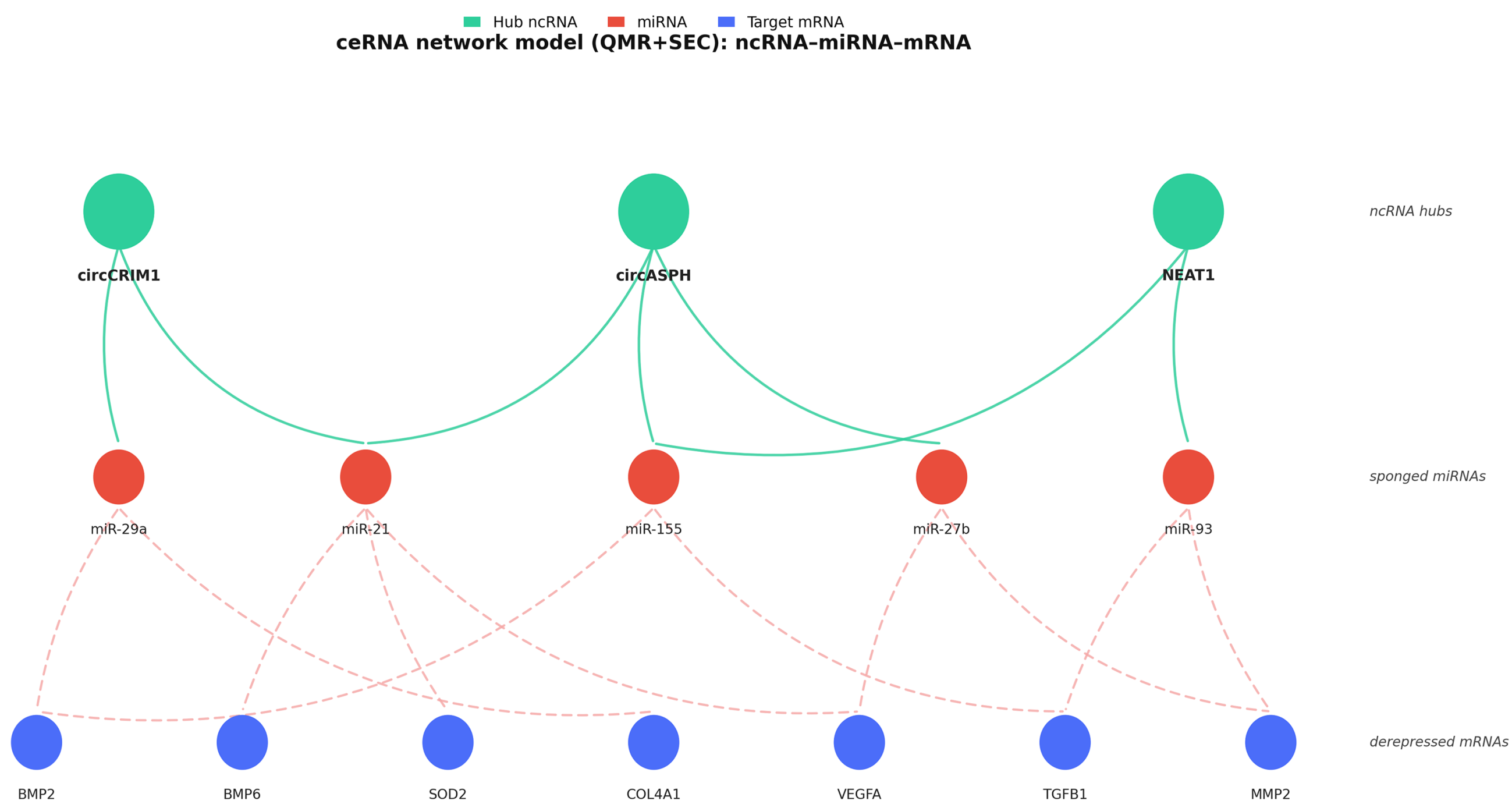

Figure 5. Master in silico ceRNA (circRNA-miRNA-mRNA) interaction network orchestrating combinatorial regenerative response

(A) Expression dynamics (time course log_2FC) of the two ultra-stable circular RNAs identified as master electrotransductive and combinatorial biomarkers. *circASPH* exhibits a massive, exclusively QMR-dependent upregulation, whereas *circCRIM1* functions as the definitive hallmark of combinatorial (QMR+SEC) combinatorial interaction. (B) Topology of the competitive endogenous RNA (ceRNA) interaction network constructed using cytoscape. The network maps the predicted functional stoichiometry, wherein the master circular and lincRNAs (large green nodes, *circCRIM1*, *circASPH*, and *NEAT1*) act as high-capacity molecular sponges, sequestering pathogenic and pro-apoptotic microRNAs (central red nodes, e.g., miR-29, miR-21, and miR-155). This sponging effect mechanistically derepresses downstream neuroprotective, anti-inflammatory, and structural target mRNAs (peripheral blue nodes), ultimately driving retinal pigment epithelium survival and extracellular matrix (ECM) homeostasis.

by the combinatory treatment. This indicates that the QMR and secretome combinatorial interaction physically remodels the structural microenvironment of the RPE, reshaping the Bruch's membrane-interface architecture—a process essential for maintaining blood-retina barrier integrity and actively preventing the structural degradation of the RPE basement membrane, which is characteristic of advanced macular dystrophies and geographic atrophy (Figure 6).

DISCUSSION

QMR as a catalyst for transcriptomic priming in RPE cells

The RPE is a highly specialized monolayer, and its structural and functional integrity is indispensable for photoreceptor survival. Its degeneration, often associated with chronic oxidative stress, is a central pathogenic event in major blinding disorders, such as AMD and RP.[37] In the present study, oxidative injury was induced in ARPE-19 cells using tBHP to model this pathological condition. While regenerative approaches have largely centered on biochemical or cell-based interventions, the effect of physical stimulation on the non-coding transcriptome of stressed RPE cells has not been previously investigated. The present data provide genome-wide evidence that QMR, a high-frequency, low-energy electromagnetic stimulus, elicits substantial yet non-cytotoxic transcriptomic reprogramming in ARPE-19 cells. PCA identified QMR as the major determinant of global transcriptomic variance, supporting the idea that QMR reconfigures the basal transcriptional landscape of the RPE. Importantly, the combined QMR+secretome treatment generated a transcriptomic profile distinct from either treatment alone, consistent with a combinatorial rather than an additive effect. These observations indicate that QMR may enhance the transcriptional responsiveness of RPE cells, thereby enabling regulatory programs that are not activated by paracrine cues alone.

Cis-regulatory linear lncRNAs mastermind lipid restructuring and oxidative defense

The cellular response to QMR and secretome is heavily stratified across distinct biotypes of the non-coding transcriptome. Linear transcripts, particularly antisense lncRNAs, have emerged as rapid-response mediators of *cis*-regulatory networks. The pervasive modulation of BASP1-AS1 and the highly significant downregulation of MFSD12-AS1 reflect precise structural and metabolic rewiring of the RPE.[1,38,39] Notably, the differential regulation of SOD2-OT1 underlines a sophisticated post-transcriptional response to oxidative stress.[40] As mitochondrial dysfunction and ROS accumulation are hallmark drivers of AMD, precise tuning of the SOD2 locus via its antisense counterpart suggests a targeted enhancement of the RPE innate antioxidant defense mechanism.[41] Furthermore, the observed

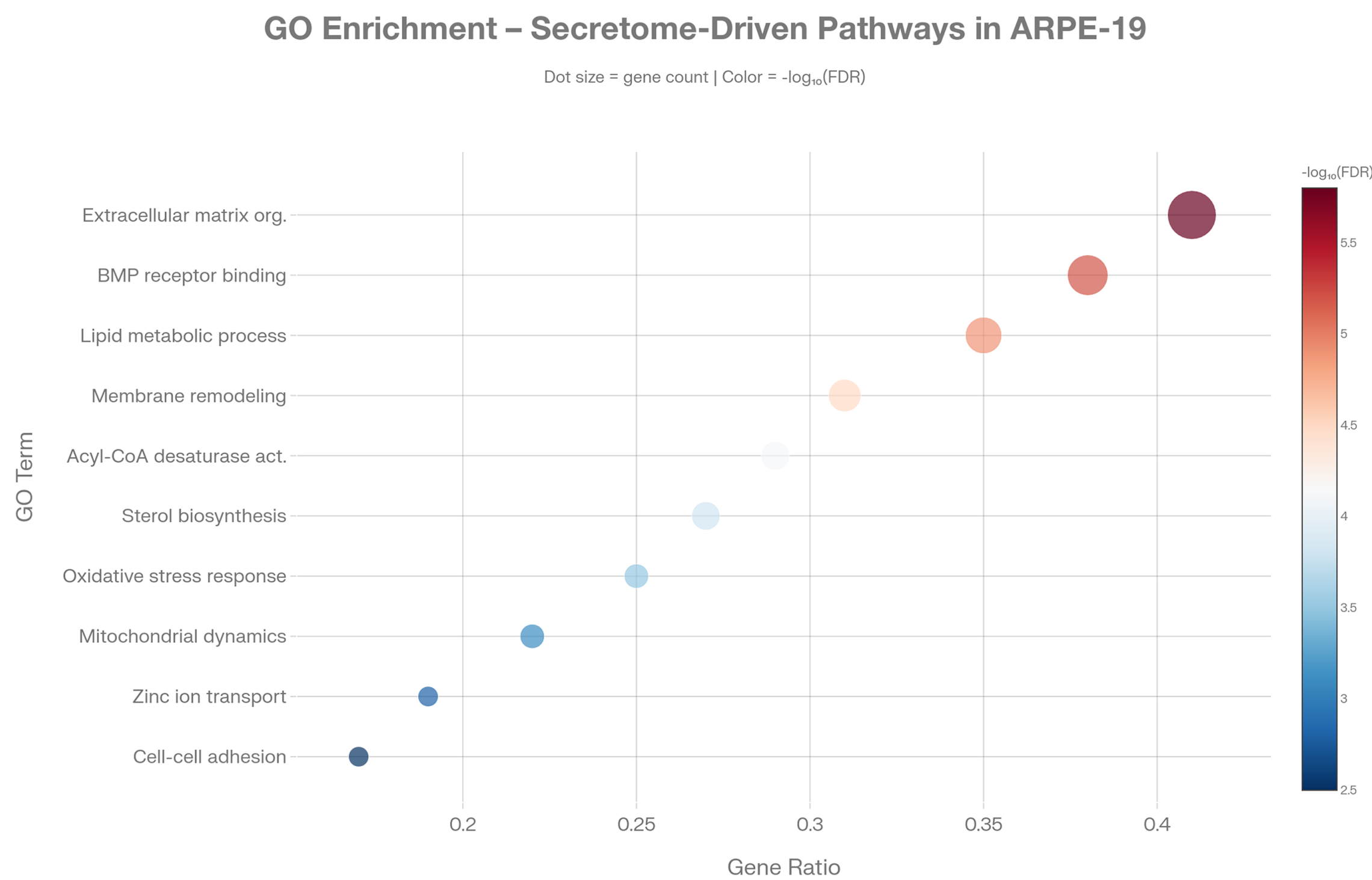

Figure 6. Functional enrichment mapping of downstream paracrine and physical effectors

(A) Gene Ontology (GO) biological process and cellular component dot plot representing the functional enrichment profile of protein-coding genes significantly correlated with the master lncRNAs modulated by the secretome. The dot size is proportional to the gene ratio within the respective ontology term, while the color gradient reflects statistical significance (FDR-adjusted *p* value). The highly enriched pathways included extracellular matrix (ECM) reorganization, lipid metabolism, and integral membrane components. (B) Mechanistic bar chart detailing the normalized expression trajectories (log_2FC) of critical neuroprotective and survival transducers, specifically highlighting the bone morphogenetic protein (BMP) signaling cascade (*BMP2*, *BMP6*) downstream of the *MALAT1* and *LINC-ROR* non-coding axes.

changes in the non-coding transcriptome perfectly aligned with the massive reorganization of lipid metabolism observed in our pathway analysis. The secretome-induced modulation of FADS2, SCD, and PCYT2, coupled with the strict suppression of cholesterol biosynthesis master genes (DHCR7, LSS, and MVK), depicts a cell actively shifting away from the pro-inflammatory drusen-forming accumulation of sterols.[42,43] Instead, ARPE-19 cells redirect their metabolic flux toward the maintenance of membrane fluidity and phospholipid turnover, a process essential for the continuous phagocytosis of photoreceptor outer segments and prevention of lipofuscin toxicity.[44,45]

Mechanotransduction and the circRNAome: *circASPH* as a sensor of physical resonance

The most significant finding of this study was the identification of a highly dynamic circRNA landscape. CircRNAs, owing to their covalently closed loop structure, are highly resistant to exonuclease degradation, making them uniquely suited to function as ultra-stable molecular sinks (ceRNAs) in post-mitotic cells, such as the RPE.[46] Our analysis identified a core ARPE-19 circRNAome, predominantly comprising highly abundant transcripts, such as *circASPH*, *circDCBLD2*, and *circCRIM1*. QMR alone was associated with a marked, specific increase in circASPH back-splice junction reads, although the very low baseline expression means the apparent fold-change should be interpreted cautiously (see limitations, translational perspectives, and future directions). The parent gene, *ASPH* (aspartate beta-hydroxylase), is intricately linked to calcium allostasis and Notch signaling.[47] We propose that *circASPH* functions as a direct electrotransductive switch in ARPE-19 cells. Upon physical stimulation by QMR, the rapid accumulation of *circASPH* acts as an immediate post-transcriptional buffer, sponging stress-induced miRNAs and preventing premature silencing of calcium-dependent survival cascades. This finding establishes circRNAs not merely as static bystanders, but as ultra-responsive elements of the physical-to-molecular transduction machinery in the retina.

A feedforward ceRNA loop: *circCRIM1* synergizes with the BMP and ECM axes

The molecular rationale for the potent therapeutic combinatorial interaction between QMR and the secretome is elegantly captured by the behavior of circCRIM1. While its baseline expression was robust, its massive upregulation was strictly contingent upon the combined QMR+SECRETOME treatment. *CRIM1* is a transmembrane regulator of BMPs.[48] Concurrently, our GO enrichment analysis independently confirmed that the secretome profoundly activates the BMP signaling axis (BMP2 and BMP6), a master pathway

in retinal neuroprotection and morphogenesis.[35,36,49] We hypothesized a therapeutic feed-forward ceRNA loop: QMR physically primes the cellular machinery to favor *CRIM1* back-splicing, and the resulting surge in *circCRIM1* transcripts acts as a highly efficient sponge for pathogenic miRNAs. Our *in silico* network construction identified high-affinity binding sites for miR-29, miR-21, and miR-155 within the sequences of our upregulated non-coding transcripts. By sequestering the pro-fibrotic miR-21, pro-inflammatory miR-155, and ECM-degrading miR-29 family, *circCRIM1* and its associated lncRNAs are predicted to derepress the BMP cascade and matrix remodelers such as ADAM12 and MMP21. This non-coding amplification mechanism sustains the neuroprotective and ECM-restructuring signals delivered by the secretome, actively restoring the Bruch's membrane-RPE interface. It must be noted that these ceRNA interactions are currently supported by in silico prediction and transcriptomic correlation data. Ongoing RNA immunoprecipitation sequencing (RIP-seq) experiments and CRISPR-Cas13-mediated knockdown of *circCRIM1*and *circASPH* are planned to provide direct functional validation of these regulatory axes in future work.

The extracellular secretome as a vector for non-coding modulators

The profound impact of the secretome on the ARPE-19 non-coding landscape invites critical mechanistic consideration of its active components. While traditional analyses of secretomes focus heavily on soluble growth factors and cytokines, our transcriptomic data strongly suggest a prominent role for EVs.[50,51] EVs are well-known carriers of complex molecular cargos, including functional lncRNAs, circRNAs, and miRNAs.[33,52] The targeted suppression of specific intronic and antisense elements (e.g., ENSG00000259661 and ENSG00000261641) following secretome application implies that paracrine EVs may deliver either competitive small RNAs that directly degrade these targets or exogenous ncRNAs that orchestrate large-scale splicing and transcriptional shifts in the recipient RPE cells. Future profiling of the secretome internal RNA cargo will be crucial for distinguishing between endogenous ARPE-19 transcriptional responses and the delivery of exogenous ncRNAs.

Canonical validation and the fingerprint of combinatorial therapy

The robustness of our multimodal therapeutic approach was further validated by the significant modulation of canonical, universally recognized "superstar" lncRNAs. The DE of *NEAT1*, *MALAT1*, *LINC-ROR*, and *RELA-DT* anchors our findings within the well-established biological paradigms of oxidative stress management, alternative splicing, and NF-κB signaling.[53–56] However, the true translational value of this study lies in the combinatorial unique molecular fingerprint generated by the QMR+SECRETOME combination. Intersection analysis formally demonstrated that the combined therapy unlocked a cadre of transcripts that remained completely dormant under single-modality treatments. This transcriptomic combinatorial interaction provides a molecular foundation for combined clinical protocols, demonstrating that merging physical and paracrine therapies yields a superior and more comprehensive regenerative response.

Limitations, translational perspectives, and future directions

While our study provides a high-resolution atlas of the ARPE-19 non-coding response to QMR and secretome therapies, several limitations warrant consideration. First, ARPE-19 is an immortalized cell line that differs substantially from primary RPE (a partially dedifferentiated transcriptome, reduced RPE65 expression, and lower phagocytic capacity); the observed non-coding responses therefore require confirmation in primary or induced pluripotent stem cell (iPSC)-derived RPE. Second, because the patient blood-derived secretome itself contains EVs and RNA cargo, we cannot exclude that some differentially expressed ncRNAs were delivered exogenously rather than induced endogenously, and profiling of the secretome RNA content is planned. Third, while our *in silico* ceRNA networks present highly candidate interactions (e.g., the *circCRIM1*-BMP axis), these require rigorous functional validation through targeted RNA pull-down assays, RIP-seq) and CRISPR-Cas13-mediated transcript-specific knockdown. Fourth, the current profiling was conducted in a highly controlled *in vitro* monolayer system; transitioning these findings to complex *in vivo* models or 3D iPSC-derived retinal organoids will be essential for evaluating the tissue-level impact on photoreceptor rescue. Ultimately, the finding that QMR and secretome can mobilize specific subclasses of the non-coding transcriptome opens promising therapeutic avenues. Highly stable circRNAs, such as *circASPH* and *circCRIM1,* have emerged as prime candidates for non-invasive ocular biomarkers in the vitreous or aqueous humor and as promising templates for the design of engineered circular antisense oligonucleotides. We advocate for the continuous expansion of a *Retinal LncRNome Atlas* to fully map the dynamic posttranscriptional programs governing retinal health and disease.

Conclusions

This study provides a comprehensive transcriptome-wide blueprint of the non-coding regulatory networks driving the ARPE-19 response to physical and paracrine therapies. Our data indicate that QMR acts as a major modulator of the ARPE-19 transcriptome, reshaping its non-coding landscape and establishing a state of increased transcriptional responsiveness. Crucially, while QMR and the secretome individually mobilize distinct, highly specialized classes of non-coding transcripts, spanning rapid *cis*-regulatory antisense lncRNAs (e.g., *SOD2-OT1*, *BASP1-AS1*) to dynamically regulated intronic elements, their combinatorial application unlocks a mathematically unique and biologically combinatorial transcriptomic signature.

At the core of this combinatorial response lies the identification of an ultra-stable circRNA interactome. The massive, condition-specific upregulation of back-spliced transcripts, such as *circASPH* (driven by physical resonance) and *circCRIM1* (driven by the combinatorial modality), establishes circRNAs as master electrotransductive sensors and high-capacity miRNA sponges in RPE cells treated with QMR. By functionally connecting these non-coding hubs to

downstream protein-coding cascades, most notably the BMP signaling axis and widespread ECM/lipid remodeling, we describe a feed-forward ceRNA loop that maximizes RPE survival and neuroprotection. These findings inform the molecular rationale for combining electroceutical and paracrine strategies for retinal dystrophies and generate hypotheses for future functional studies. The identified condition-specific lncRNAs and circRNAs are promising candidates for non-invasive ocular biomarkers and represent specific targets for next-generation RNA-based therapeutics (such as engineered circular antisense oligonucleotides) aimed at halting the progression of AMD and RP.

MATERIALS AND METHODS

Cell culture

Human retinal pigment epithelial ARPE-19 cells (ATCC CRL-2302; American Type Culture Collection, Manassas, VA, USA) were used as an *in vitro* experimental model. Cells were cultured in high-glucose DMEM/F-12 (Thermo Fisher Scientific, Waltham, MA, USA; Cat. no. 11320-033) supplemented with 10% fetal bovine serum, 2 mM L-glutamine, and 100 U/mL penicillin-streptomycin, and maintained at 37°C in a humidified incubator with 5% CO2 concentration. Cell-line identity was validated by short tandem repeat analysis with the PowerPlex 10 System (Promega Corporation, Madison, WI, USA), showing a short tandem repeat profile concordant with the ARPE-19 reference (PowerPlex 10 System; 8 of 9 core short tandem repeats (STR) loci matched the ATCC ARPE-19 reference; approximately 89% allele concordance). Mycoplasma contamination was routinely excluded every two months using the MycoAlert PLUS kit (Lonza Group AG, Basel, Switzerland).

Oxidative stress induction

To model the degenerative environmental characteristics of retinal dystrophies, oxidative stress was induced in ARPE-19 cells prior to treatment. Cells were exposed to (tBHP; Sigma-Aldrich, St. Louis, MO, USA), a well-established inducer of oxidative damage in RPE cells.[57] A stock solution of tBHP was prepared in the culture medium and applied to the cells at a final concentration of 75 μM, empirically determined in preliminary MTT (3-(4,5-dimethylthiazol-2-yl)-2,5-diphenyltetrazolium bromide) titrations to reduce cell viability by approximately 50% after 24 h, as assessed by preliminary MTT assays (see the cell viability assay). Cells were treated with tBHP starting at time 0 and continuously maintained under oxidative stress for the entire experimental period (8, 24, and 72 h). This approach ensures that subsequent treatments with QMR and the secretome are evaluated in the biologically relevant context of oxidative injury, mimicking key aspects of RPE degeneration.

QMR and secretome treatments

Physical stimulation was delivered using a benchtop QMR generator (Telea Electronic Engineering srl, Sandrigo, Italy; 230 V, 50/60 Hz, max 250 VA). The device emitted multifrequency signals comprising a fundamental frequency of 4 MHz and higher-order harmonics ranging from 8 to 64 MHz. For the *in vitro* setup, two flat electrodes, both made of surgical-grade stainless steel (AISI 316 L), were used. They were inserted at the edge of the 90 mm plate until they contacted the medium surface. The culture medium volume was strictly maintained at 12 mL to yield an estimated electric field strength of 1.1 ± 0.2 V/cm at 2 mm above the monolayer (validated via Ag/AgCl microelectrodes, n = 3), resulting in a current density of $\sim$12 mA/cm^2. Each QMR session lasted 10 min at a nominal power setting of 40 (≈ 10 W output). We note that “QMR” is a commercial designation for a radiofrequency electromagnetic-field device operating at 4–64 MHz; the term “quantum” does not imply that quantum-mechanical effects are the operative mechanism. For the secretome treatment group, ARPE-19 cells were cultured in medium supplemented with a patient blood-derived secretome (platelet-rich-plasma releasate) applied at a final concentration of 50% (v/v); this secretome was obtained from the peripheral blood of a single adult donor, activated with CaCl2 (20–25 mM), 0.22-μm filtered, concentrated by 3-kDa ultrafiltration, quantified by bicinchoninic acid assay (BCA), and contains soluble growth factors (e.g., VEGF, TGF-β1, platelet-derived growth factor [PDGF], insulin growth factor [IGF]-1), cytokines, and EVs, derived according to our previously standardized protocols (approved by the Ethics Committee of A.O.U. "G. Martino" of Messina, with n° 858 of June 23, 2017).[57] To evaluate temporal dynamics and potential synergies, the experimental design included four distinct groups: CTRL) QMR alone, secretome alone (SEC), and combinatorial treatment (QMR+SECRETOME). For the 8 and 24 h experimental time points, cells assigned to the physical and combination treatments (QMR and QMR+SECRETOME) received a single 10-min QMR session at t = 0 h. At the 72 h time point, to evaluate cumulative effects, cells received three consecutive 10-min sessions at t = 0, ∼24, and ∼48 h. Sham-treated CTRL underwent identical plate handling, electrode positioning, and incubation intervals with the QMR device powered off, ensuring rigorously blinded handling conditions throughout all the procedures.

Cell viability assay

Cell viability across all experimental conditions was monitored at the specified time points using the MTT reduction assay (Sigma-Aldrich, St. Louis, MO, USA). Following treatment, the cells were incubated with MTT solution (5 mg/mL in PBS) for 2 h at 37°C. Insoluble formazan crystals were subsequently dissolved using 10% SDS in 0.01 mol/L HCl overnight. Absorbance was measured at 570 nm using a microplate reader. Viability was expressed as a percentage normalized to that of the untreated CTRL group.

Total RNA extraction and sequencing

Total RNA was extracted from the 72 ARPE-19 samples using TRIzol Reagent (Invitrogen, Thermo Fisher Scientific, Waltham, MA, USA) according to the manufacturer’s instructions. RNA concentration and integrity were quantified using a Qubit 2.0 fluorometer (Invitrogen) and an Agilent Bioanalyzer (RIN ≥8 for all samples). Sequencing libraries were generated from 1 μg of total RNA using the TruSeq Stranded Total RNA Sample Prep Kit with Ribo-Zero HMR (Illumina, San Diego, CA, USA) to efficiently deplete ribosomal RNA while preserving both coding and non-coding

transcripts. High-depth paired-end sequencing was performed on an Illumina NovaSeq 6000 platform to a target depth of at least 30 million paired-end reads per sample (libraries failing Q30 ≥ 80% were excluded; per-sample read counts are reported in Figure S1).

Quality assessment, read alignment, and quantification

Raw sequencing reads were subjected to rigorous quality control using FastQC (v.0.11.9) and QualiMap (v.2.2.1). Adapter sequences and low-quality bases (average Phred score <30) were removed using Trimmomatic (v.0.39). The filtered reads were aligned against the human reference genome (GRCh38/hg38) and the comprehensive Ensembl non-coding RNA database (v.109) using Qiagen CLC Genomics Workbench (v.26.0.0) with the following local-alignment settings: length fraction 0.8, similarity fraction 0.8, mismatch cost 2, insertion/deletion cost 3, and a maximum of 10 hits per read. Transcript quantification used an alignment-dependent expectation maximization (EM) algorithm to distribute multi-mapping reads among highly similar transcripts. Transcript abundances were expressed as TPM for within-sample, length-normalized visualization; for DE, raw counts were normalized between samples using the trimmed mean of *M* values (TMM) method (calcNormFactors) prior to the Limma-voom transformation. TPM and TMM therefore served distinct and complementary purposes (within-sample, length-based abundance scaling versus between-sample library-size/composition normalization).

Stringent filtering and annotation of linear lncRNA classes

To isolate the long non-coding transcriptome, mapped reads were strictly filtered based on structural and expression criteria: sequence length >200 nucleotides and a minimum normalized count of TPM ≥1 across replicates. The extracted pool was subjected to comprehensive annotation and sub-classification (e.g., antisense, lincRNA, sense-intronic, sense-overlapping, and bidirectional/pancRNA) by integrating data from GENCODE (v.45), LNCipedia (v.6), RNAinter, and lncRNADisease (v.3). To eliminate transcripts with hidden protein-coding capacity, alignment-free algorithms, including the coding-potential assessment tool (CPAT), CNCI, PLEK, and FEELnc, were applied. Transcripts classified as "to be experimentally confirmed" (TEC) by Ensembl were retained for exploratory analyses.

circRNA detection and back-splice junction mapping

To comprehensively profile the circular non-coding transcriptome, raw RNA-seq reads spanning non-collinear back-splice junctions were isolated from the samples. Given their unique topology, circRNAs were detected using a multi-algorithm, high-stringency pipeline to minimize the number of false positives. Unmapped reads were processed using a pseudo-reference-based tool (KNIFE, 2015 release) and chiastic-clipping back-splice detection algorithms (CIRCexplorer2 v.2.3.8 and CIRI2 v.2.0.6) to identify back-splicing events. Only circRNAs supported by at least two independent back-spliced junction reads in each of at least two biological replicates, and called concordantly by at least two of the three algorithms (consensus calling), were retained; discordant single-tool calls were discarded. The putative circularity of the prioritized candidates (circASPH, circCRIM1) will be confirmed by RNase R-resistance assays in ongoing validation experiments. The genomic coordinates of the validated back-splices were mapped against the GRCh38 genome to precisely annotate the corresponding host genes.

DE analysis and principal component segregation

DE analysis of the non-coding and protein-coding transcriptomes was performed using the Limma R package with the voom transformation. General linear models were constructed to compare expression shifts across six full factorial pairwise contrasts: QMR vs. CTRL, SEC vs. CTRL, QMR+SEC vs. CTRL, SEC vs. QMR, QMR+SEC vs. QMR, and SEC vs. QMR+SEC. A multiple-group mean comparison (due to treatment effect) was also calculated. Significance was determined using the empirical Bayes moderated *t* test, with *p* values adjusted using the Benjamini-Hochberg (BH) method to control the FDR. Transcripts exhibiting an FDR <0.05 and an absolute $\log_2$ fold change ($|\log_2 FC|$) > 1 were considered highly significantly differentially expressed. PCA and unsupervised hierarchical clustering (Euclidean distance, Ward's linkage) were performed on normalized *Z* scores to visualize global sample segregation and construct feature-level heatmaps.

Pathway enrichment and ceRNA network construction

To elucidate the functional consequences of transcriptomic reprogramming, GO enrichment analysis (MF, CC, and BP) was performed on the predicted protein-coding targets of the differentially expressed ncRNAs, defined as experimentally supported or high-confidence predicted miRNA targets within the ceRNA network (miRNet 2.0, DIANA-LncBase v.3, CircInteractome) and restricted to genes detected (TPM ≥1) in our dataset. Significance was set at an FDR-adjusted *p* value of <0.05. For the construction of ceRNA networks, the potential of the top differentially expressed lncRNAs and circRNAs to act as miRNA sponges was assessed. Sequence-based miRNA target prediction was performed using miRNet 2.0, DIANA-LncBase (v.3), and CircInteractome, focusing exclusively on highly conserved, high-affinity miRNA binding sites (e.g., hsa-miR-29, hsa-miR-155, and hsa-miR-21–5p) identified within the upregulated non-coding transcripts.

DATA AND CODE AVAILABILITY

The raw RNA-seq data generated in this study have been deposited in the NCBI Gene Expression Omnibus (GEO) under accession number GSE328981. All processed data supporting the findings are available from the corresponding author upon reasonable request.

ACKNOWLEDGMENTS

This research received no external funding. Experiments using the commercially available ARPE-19 cell line did not require ethics approval; the patient blood-derived secretome was prepared under protocols approved by the Ethics Committee of A.O.U. G. Martino (approval no. 858, June 23, 2017), and written informed consent was obtained from all blood donors.

AUTHOR CONTRIBUTIONS

Conceptualization, L.D. and S.A.; methodology and investigation, S.A.; software, formal analysis, writing – original draft preparation, and project administration, L.D.;

validation, D.M. and S.A.; resources, G.P. and A.D.A.; data curation, R.D.; writing – review and editing, S.A., G.A., and D.M.; visualization, C.S., R.D., and F.A.; supervision, A.S.; funding acquisition, L.D. and S.Z.S. All authors have read and agreed to the published version of the manuscript.

DECLARATION OF INTERESTS

G.P. is an employee and scientific director of Telea Electronic Engineering Srl (Sandrigo, Italy), the company that manufactures and commercializes the QMR device used in this study. His role was limited to providing the QMR device and its technical specifications; Telea Electronic Engineering had no role in the design of the study, in the collection, analysis, or interpretation of the data, in the writing of the manuscript, or in the decision to publish the results. All data generation, bioinformatic and statistical analysis, and interpretation were performed exclusively by the academic co-authors (University of Messina and I.E.ME.S.T.), who have no commercial interest in the outcome.

SUPPLEMENTAL INFORMATION

Supplemental information can be found online at https://doi.org/10.1016/j.omtn.2026.103034.

REFERENCES

1. Miller, R.D., Mondon, I., Ellis, C., Muir, A.-M., Turner, S., Keeling, E., Wai, H.A., Chatelet, D.S., Johnson, D.A., Tumbarello, D.A., et al. (2025). Whole RNA-Seq Analysis Reveals Longitudinal Proteostasis Network Responses to Photoreceptor Outer Segment Trafficking and Degradation in RPE Cells. Cells *14*, 1166. https://doi.org/10.3390/cells14151166.
2. Kafida, M., Karela, M., and Giakountis, A. (2024). RNA-Independent Regulatory Functions of lncRNA in Complex Disease. Cancers *16*, 2728. https://doi.org/10.3390/cancers16152728.
3. Kaur, G., Perteghella, T., Carbonell-Sala, S., Gonzalez-Martinez, J., Hunt, T., Mądry, T., Jungreis, I., Arnan, C., Lagarde, J., Borsari, B., et al. (2024). GENCODE: massively expanding the lncRNA catalog through capture long-read RNA sequencing. Preprint at bioRxiv. https://doi.org/10.1101/2024.10.29.620654.
4. Lamping, J.-P., and Krebber, H. (2025). The hidden power of antisense long non-coding RNAs: a dive into a novel regulatory layer mediated by double-stranded RNA formation. RNA Biol. *22*, 1–16. https://doi.org/10.1080/15476286.2025.2530797.
5. Kyung, J., Kim, M., Shin, H.-R., Kim, E., and Oh, H.J. (2026). Multi-layered gene regulation by long non-coding RNAs: from chromatin to genome architecture. BMB Rep. *59*, 112–123. https://doi.org/10.5483/BMBRep.2025-0176.
6. Carranza, F., Shenasa, H., and Hertel, K.J. (2022). Splice site proximity influences alternative exon definition. RNA Biol. *19*, 829–840. https://doi.org/10.1080/15476286.2022.2089478.
7. An, B., Kameda, T., and Imamura, T. (2021). The evolutionary acquisition and mode of functions of promoter-associated non-coding RNAs (pancRNAs) for mammalian development. Essays Biochem. *65*, 697–708. https://doi.org/10.1042/ebc20200143.
8. Baiddou, C., Knidiri, M., Ghazi, B., Abu Obaid, A., Hamdi, S., and Errafii, K. (2026). The circular RNA landscape: Biogenesis, functions, identification pipelines, and biomedical applications. Noncoding RNA Res. *18*, 39–51. https://doi.org/10.1016/j.ncrna.2025.12.005.
9. Sun, L.F., Chen, X.J., and Jin, Z.B. (2020). Emerging roles of non-coding RNAs in retinal diseases: A review. Clin. Exp. Ophthalmol. *48*, 1085–1101. https://doi.org/10.1111/ceo.13806.
10. Kaczynski, T.J., Au, E.D., and Farkas, M.H. (2022). Exploring the lncRNA localization landscape within the retinal pigment epithelium under normal and stress conditions. BMC Genom. *23*, 539. https://doi.org/10.1186/s12864-022-08777-1.
11. Wang, X., Zhuang, H., Dang, Y., and Lei, F. (2025). Transcriptome Sequencing Reveals ceRNA Networks and Molecular Signatures in Myopic Mouse Retina. Curr. Eye Res. *50*, 1181–1194. https://doi.org/10.1080/02713683.2025.2541448.
12. Wubben, T.J., Weh, E., and Besirli, C.G. (2025). Photoreceptor Degeneration: More Than a Bystander in Age-Related Macular Degeneration. Adv. Exp. Med. Biol. *1468*, 27–31. https://doi.org/10.1007/978-3-031-76550-6_5.
13. Ghanawi, H., and Koch, S.F. (2025). The versatile roles of retinal pigment epithelium in the pathophysiology of retinitis pigmentosa. Prog. Retin. Eye Res. *108*, 101390. https://doi.org/10.1016/j.preteyeres.2025.101390.
14. Chen, X., Jiang, C., Qin, B., Liu, G., Ji, J., Sun, X., Xu, M., Ding, S., Zhu, M., Huang, G., et al. (2017). LncRNA ZNF503-AS1 promotes RPE differentiation by downregulating ZNF503 expression. Cell Death Dis. *8*, e3046. https://doi.org/10.1038/cddis.2017.382.
15. An, H., Williams, N.G., and Shelkovnikova, T.A. (2018). NEAT1 and paraspeckles in neurodegenerative diseases: A missing lnc found? Noncoding RNA Res. *3*, 243–252. https://doi.org/10.1016/j.ncrna.2018.11.003.
16. Yang, G., and Huang, X. (2025). LncRNA MALAT1's role in the development of retinopathy: A review. Medicine *104*, e41954. https://doi.org/10.1097/md.0000000000041954.
17. Wang, S., Jiang, W., Zhang, X., Lu, Z., Geng, Q., Wang, W., Li, N., and Cai, X. (2020). LINC-PINT alleviates lung cancer progression via sponging miR-543 and inducing PTEN. Cancer Med. *9*, 1999–2009. https://doi.org/10.1002/cam4.2822.
18. Zhang, A., Zhou, N., Huang, J., Liu, Q., Fukuda, K., Ma, D., Lu, Z., Bai, C., Watabe, K., and Mo, Y.-Y. (2013). The human long non-coding RNA-RoR is a p53 repressor in response to DNA damage. Cell Res. *23*, 340–350. https://doi.org/10.1038/cr.2012.164.
19. Sharma, A., and Singh, N.K. (2023). Long Non-Coding RNAs and Proliferative Retinal Diseases. Pharmaceutics *15*, 1454. https://doi.org/10.3390/pharmaceutics15051454.
20. Barros Ferreira, L., Ashander, L.M., Appukuttan, B., Ma, Y., Williams, K.A., and Smith, J.R. (2023). Expression of Long Non-Coding RNAs in Activated Human Retinal Vascular Endothelial Cells. Ocul. Immunol. Inflamm. *31*, 1813–1818. https://doi.org/10.1080/09273948.2022.2122512.
21. Donato, L., Scimone, C., Alibrandi, S., Rinaldi, C., Sidoti, A., and D'Angelo, R. (2020). Transcriptome Analyses of lncRNAs in A2E-Stressed Retinal Epithelial Cells Unveil Advanced Links between Metabolic Impairments Related to Oxidative Stress and Retinitis Pigmentosa. Antioxidants *9*, 318. https://doi.org/10.3390/antiox9040318.
22. Feng, Y., Yang, F., Zheng, J., Shi, L., Xie, T., Lin, Y., and Shi, Q. (2024). Circular RNA HIPK3 mediates epithelial–mesenchymal transition of retinal pigment epithelial cells by sponging multiple microRNAs. Sci. Rep. *14*, 28872. https://doi.org/10.1038/s41598-024-71119-6.
23. Donato, L., D'Angelo, R., Alibrandi, S., Rinaldi, C., Sidoti, A., and Scimone, C. (2020). Effects of A2E-Induced Oxidative Stress on Retinal Epithelial Cells: New Insights on Differential Gene Response and Retinal Dystrophies. Antioxidants *9*, 307. https://doi.org/10.3390/antiox9040307.
24. Scimone, C., Alibrandi, S., Scalinci, S.Z., Trovato Battagliola, E., D'Angelo, R., Sidoti, A., and Donato, L. (2020). Expression of Pro-Angiogenic Markers Is Enhanced by Blue Light in Human RPE Cells. Antioxidants *9*, 1154. https://doi.org/10.3390/antiox9111154.
25. Donato, L., Scimone, C., Alibrandi, S., Scalinci, S.Z., Rinaldi, C., D'Angelo, R., and Sidoti, A. (2022). Epitranscriptome Analysis of Oxidative Stressed Retinal Epithelial Cells Depicted a Possible RNA Editing Landscape of Retinal Degeneration. Antioxidants *11*, 1967. https://doi.org/10.3390/antiox11101967.
26. Donato, L., Scimone, C., Alibrandi, S., Pitruzzella, A., Scalia, F., D'Angelo, R., and Sidoti, A. (2020). Possible A2E Mutagenic Effects on RPE Mitochondrial DNA from Innovative RNA-Seq Bioinformatics Pipeline. Antioxidants *9*, 1158. https://doi.org/10.3390/antiox9111158.
27. Canós-Verdecho, Á., Robledo, R., Izquierdo, R., Bermejo, A., Gallach, E., Argente, P., Peraita-Costa, I., and Morales-Suárez-Varela, M. (2022). Preliminary evaluation of the efficacy of quantum molecular resonance coablative radiofrequency and microdiscectomy. Pain Manag. *12*, 917–930. https://doi.org/10.2217/pmt-2022-0039.
28. Dieper, A., Scheidegger, S., Füchslin, R.M., Veltsista, P.D., Stein, U., Weyland, M., Gerster, D., Beck, M., Bengtsson, O., Zips, D., and Ghadjar, P. (2024). Literature review: potential non-thermal molecular effects of external radiofrequency electromagnetic fields on cancer. Int. J. Hyperthermia *41*, 2379992. https://doi.org/10.1080/02656736.2024.2379992.

29. Szeto, H.H. (2018). Stealth Peptides Target Cellular Powerhouses to Fight Rare and Common Age-Related Diseases. PPL *25*, 1108–1123. https://doi.org/10.2174/0929866525666181101105209.

30. Ratajczak, M.Z., Kucia, M., Jadczyk, T., Greco, N.J., Wojakowski, W., Tendera, M., and Ratajczak, J. (2012). Pivotal role of paracrine effects in stem cell therapies in regenerative medicine: can we translate stem cell-secreted paracrine factors and microvesicles into better therapeutic strategies? Leukemia *26*, 1166–1173. https://doi.org/10.1038/leu.2011.389.

31. Uhlén, M., Karlsson, M.J., Hober, A., Svensson, A.-S., Scheffel, J., Kotol, D., Zhong, W., Tebani, A., Strandberg, L., Edfors, F., et al. (2019). The human secretome. Sci. Signal. *12*, eaaz0274. https://doi.org/10.1126/scisignal.aaz0274.

32. Tüshaus, J., Müller, S.A., Kataka, E.S., Zaucha, J., Monasor, L.S., Su, M., Güner, G., Jocher, G., Tahirovic, S., Frishman, D., et al. (2020). Quantitative secretome analysis establishes the cell type-resolved mouse brain secretome. EMBO J. *39*, e105693. https://doi.org/10.1101/2020.05.22.110023.

33. Abdelgawad, A., Huang, Y., Gololobova, O., Yu, Y., Witwer, K.W., Parashar, V., and Batish, M. (2025). Defining the Parameters for Sorting of RNA Cargo Into Extracellular Vesicles. J. Extracell. Vesicles *14*, e70113. https://doi.org/10.1002/jev2.70113.

34. Fang, C., He, D., Qian, Y., and Shen, X. (2025). BMP4-GPX4 can improve the ferroptosis phenotype of retinal ganglion cells and enhance their differentiation ability after retinal stem cell transplantation in glaucoma with high intraocular pressure. Hum. Mol. Genet. *34*, 673–683. https://doi.org/10.1093/hmg/ddaf011.

35. Steinfeld, J., Steinfeld, I., Bausch, A., Coronato, N., Hampel, M.-L., Depner, H., Layer, P.G., and Vogel-Höpker, A. (2017). BMP-induced reprogramming of the neural retina into retinal pigment epithelium requires Wnt signalling. Biol. Open *6*, 979–992. https://doi.org/10.1242/bio.018739.

36. Wordinger, R.J., and Clark, A.F. (2007). Bone Morphogenetic Proteins and Their Receptors in the Eye. Exp. Biol. Med. *232*, 979–992. https://doi.org/10.3181/0510-mr-345.

37. Markitantova, Y., and Simirskii, V. (2025). Retinal Pigment Epithelium Under Oxidative Stress: Chaperoning Autophagy and Beyond. IJMS *26*, 1193. https://doi.org/10.3390/ijms26031193.

38. Yang, C., Georgiou, M., Atkinson, R., Collin, J., Al-Aama, J., Nagaraja-Grellscheid, S., Johnson, C., Ali, R., Armstrong, L., Mozaffari-Jovin, S., and Lako, M. (2021). Pre-mRNA Processing Factors and Retinitis Pigmentosa: RNA Splicing and Beyond. Front. Cell Dev. Biol. *9*, 700276. https://doi.org/10.3389/fcell.2021.700276.

39. Trifunovic, D., Karali, M., Camposampiero, D., Ponzin, D., Banfi, S., and Marigo, V. (2008). A high-resolution RNA expression atlas of retinitis pigmentosa genes in human and mouse retinas. Investig. Ophthalmol. Vis. Sci. *49*, 2330–2336. https://doi.org/10.1167/iovs.07-1513.

40. Damiano, S., Sozio, C., La Rosa, G., Guida, B., Faraonio, R., Santillo, M., and Mondola, P. (2020). Metabolism Regulation and Redox State: Insight into the Role of Superoxide Dismutase 1. IJMS *21*, 6606. https://doi.org/10.3390/ijms21186606.

41. Mimura, T., and Noma, H. (2025). Oxidative Stress in Age-Related Macular Degeneration: From Molecular Mechanisms to Emerging Therapeutic Targets. Antioxidants *14*, 1251. https://doi.org/10.3390/antiox14101251.

42. Sun, Q., Xing, X.-R., Liu, C., Jia, D.-D., and Wang, R. (2025). Advances in the function and mechanisms of stearoyl-CoA desaturase 1 in metabolic diseases. Sheng Li Xue Bao *77*, 545–562. https://doi.org/10.13294/j.aps.2025.0001.

43. Shetty, V.V., and Shetty, S.S. (2025). Fatty Acid Desaturase: The Yin or Yang of Disease Pathology. Mol. Diagn. Ther. *29*, 637–654. https://doi.org/10.1007/s40291-025-00796-4.

44. Finnemann, S.C., Leung, L.W., and Rodriguez-Boulan, E. (2002). The lipofuscin component A2E selectively inhibits phagolysosomal degradation of photoreceptor phospholipid by the retinal pigment epithelium. Proc. Natl. Acad. Sci. USA *99*, 3842–3847. https://doi.org/10.1073/pnas.052025899.

45. Etchegaray, J.I., and Ravichandran, K. (2025). Role of RPE Phagocytosis in the Retina Metabolic Ecosystem. Adv. Exp. Med. Biol. *1468*, 429–433. https://doi.org/10.1007/978-3-031-76550-6_70.

46. Tian, H., Zhao, L., Li, H., Huang, Y., and Wang, Y. (2023). Circular RNA in Retina: A Potential Biomarker and Therapeutic Target. Ophthalmic Res. *66*, 516–528. https://doi.org/10.1159/000529207.

47. Tricarico, P.M., and Crovella, S. (2023). Notch Signaling in Health and Disease. IJMS *24*, 16113. https://doi.org/10.3390/ijms242216113.

48. Wilkinson, L., Kolle, G., Wen, D., Piper, M., Scott, J., and Little, M. (2003). CRIM1 Regulates the Rate of Processing and Delivery of Bone Morphogenetic Proteins to the Cell Surface. J. Biol. Chem. *278*, 34181–34188. https://doi.org/10.1074/jbc.m301247200.

49. Liu, X., Liu, M., and Chen, L. (2024). Bone morphogenetic protein 6 (BMP6) antagonises experimental proliferative vitreoretinopathy established by TGF-β2 stimulation in retinal pigment epithelial cells through modulation of the p38 and JNK MAPK pathways. Cell Tissue Res. *396*, 103–117. https://doi.org/10.1007/s00441-024-03870-1.

50. Schank, M.B., Zhao, J., Wang, L., Moorman, J.P., and Yao, Z.Q. (2026). Extracellular Vesicles: A Comprehensive Review of Their Origins, Functions, and Therapeutic Potential. Biomedicines *14*, 495. https://doi.org/10.3390/biomedicines14030495.

51. Waqas, M.Y., Javid, M.A., Nazir, M.M., Niaz, N., Nisar, M.F., Manzoor, Z., Bhatti, S.A., Hameed, S., and Khaliq, M.H. (2022). Extracellular vesicles and exosome: insight from physiological regulatory perspectives. J. Physiol. Biochem. *78*, 573–580. https://doi.org/10.1007/s13105-022-00877-6.

52. Luo, Y.-W., Liu, C.-G., Kirby, J.A., Chu, C., Zang, D., and Chen, J. (2025). The Emerging Role of Extracellular Vesicle-Derived lncRNAs and circRNAs in Tumor and Mesenchymal Stem Cells: The Biological Functions and Potential for Clinical Application. Cancers *17*, 2186. https://doi.org/10.3390/cancers17132186.

53. Luo, Z., Yi, Z.J., Ou, Z.L., Han, T., Wan, T., Tang, Y.C., Wang, Z.C., and Huang, F.Z. (2019). RELA/NEAT1/miR-302a-3p/RELA feedback loop modulates pancreatic ductal adenocarcinoma cell proliferation and migration. J. Cell. Physiol. *234*, 3583–3597. https://doi.org/10.1002/jcp.27039.

54. Ghafouri-Fard, S., Pourtavakoli, A., Hussen, B.M., Taheri, M., and Kiani, A. (2023). A review on the importance of LINC-ROR in human disorders. Pathol. Res. Pract. *244*, 154420. https://doi.org/10.1016/j.prp.2023.154420.

55. Poller, C., Kuss, A.W., Weiss, S., Haghikia, A., Gast, M., Mochmann, L., Zeller, T., Blankenberg, S., Felix, S., Voelker, U., et al. (2020). Experimental and population-genetic evidence for inflammation control functions of long noncoding RNAs and a novel tRNA-like transcript arising from the human NEAT1-MALAT1 genomic region. Eur. Heart J. *41*, ehaa946.3759. https://doi.org/10.1093/ehjci/ehaa946.3759.

56. De Domenico, S., La Banca, V., D'Amico, S., Nicolai, S., and Peschiaroli, A. (2025). Defining the transcriptional routes controlling lncRNA NEAT1 expression: implications in cellular stress response, inflammation, and differentiation. Discov Onc *16*, 768. https://doi.org/10.1007/s12672-025-02510-6.

57. Alibrandi, S., Mordà, D., Scimone, C., D'Ascola, A., Aliquò, F., Pozzato, A., Scalinci, S.Z., D'Angelo, R., Sidoti, A., and Donato, L. (2025). QMR® and Patient Blood-Derived Secretome Modulate RPE microRNA Networks Under Oxidative Stress. Int. J. Mol. Sci. *26*, 8614. https://doi.org/10.3390/ijms26178614.

OMTN, Volume *37*

Supplemental information

Quantum molecular resonance and secretome synergistically unlocks a protective ceRNA axis in the human retinal pigment epithelium

Simona Alibrandi, Domenico Mordà, Concetta Scimone, Angela D'Ascola, Federica Aliquò, Giorgia Abate, Gianantonio Pozzato, Sergio Zaccaria Scalinci, Rosalia D'Angelo, Antonina Sidoti, and Luigi Donato

Supplemental Material

Supplemental Tables

Table S1. Complete differential expression statistics across six full-factorial experimental contrasts. This master table provides global differential expression profiling of all detected non-coding transcripts. For each of the six pairwise comparisons (QMR vs. CTRL, SECRETOME vs. CTRL, QMR+SEC vs. CTRL, QMR+SEC vs. QMR, QMR+SEC vs. SEC, and SEC vs. QMR), the table reports the corresponding $\log_2$ Fold Change ($\log_2$FC) and False Discovery Rate (FDR/adjusted p-value). Transcripts that met the rigorous significance thresholds (FDR < 0.05, $|\log_2FC| > 1$) were flagged for each specific contrast.

Provided as a separate Excel file: Table_S1_Differential_Expression_Complete.xlsx.

Table S2. Comprehensive inventory of the ARPE-19 non-coding transcriptome. Complete catalog of the 2,313 detected non-coding transcripts (TPM > 0.1) across all 72 biological samples. The dataset included Ensembl IDs, assigned gene symbols, exact genomic coordinates (chromosome, start, end, and strand), exon counts, and transcript biotype classifications (e.g., antisense, lincRNA, sense-intronic, and circRNA). Mean Transcripts Per Kilobase Million (TPM) values were provided for the CTRL, QMR, SECRETOME, and QMR+SECRETOME experimental groups to establish baseline expression levels.

Provided as a separate Excel file: Table_S2_All_Detected_lncRNAs.xlsx.

Table S3. Gene Ontology (GO) functional enrichment analysis of differentially expressed non-coding networks. The comprehensive output of the pathway enrichment analysis derived from the protein-coding targets strongly correlated with the master ncRNAs. The table categorizes significantly enriched terms (FDR < 0.05) into three primary GO ontologies: Biological Process (BP), Molecular Function (MF), and Cellular Component (CC). For each enriched term, the table details the GO ID, biological description, number of mapped genes, Gene Ratio, exact FDR-adjusted p-value, and specific gene set driving the enrichment (e.g., BMP2, BMP6, and FADS2).

Provided as a separate Excel file: Table_S3_GO_Enrichment_Complete.xlsx.

Table S4. Oligonucleotide primer sequences designed for the qRT-PCR validation of prioritized non-coding transcripts. The table reports the sequence features (Forward and Reverse primers, 5'→3' orientation) and expected amplicon size for the top 10 significantly modulated ncRNAs selected for technical validation. GAPDH and ACTB were used as internal endogenous reference controls for normalization. For circular RNAs, circASPH and circCRIM1, specifically designed divergent primers spanning the unique non-canonical back-splice junction were employed to prevent co-amplification of linear host transcripts.

Gene Symbol	Primer Direction (5'_3')	Sequence (5'_3')	Expected Amplicon Size (bp)
circASPH(divergent)	Forward	GCCAAGATCTGAGGCCTACC	148
	Reverse	CTCAGGTTCTTGGCCACATC	
circCRIM1(divergent)	Forward	TGTGTCCTGGCTACTGTGTC	135
	Reverse	ACAGCAGTAGCTCCCACATC	
NEAT1	Forward	TGGCTAGCTCAGGGCTTCAG	172
	Reverse	TCTCCTTGCCAAGCTTCCTTC	
BASP1-AS1	Forward	GGCATCCAGACACAGCATTC	155
	Reverse	CCCTGGAGCTCCAAACATCA	
MALAT1	Forward	AAAGCAAGGTCTCCCCACAAG	129
	Reverse	GGTCTGTGCTAGATCAAAAGGCA	
TAPBP-AS1	Forward	TCTGGCCCATGTCACAAAGT	160
	Reverse	GGCCTCAGCCTCTCTTTAGC	
LINC-ROR	Forward	AGGAAGCCTGAGAGTTGGC	185
	Reverse	CTCAGTGGGGAAGACTCCAG	
SOD2-OT1	Forward	GGACACGAAGAATCTGAGGC	142
	Reverse	CTTCAGCATTGCCCTTACCC	
RELA-DT	Forward	CCTGGGATCAGATGTGTGGT	150
	Reverse	TCTCAGCCCTGGATTTTGCG	
CSRNP1-AS1	Forward	AGCACAGAGCAGAAGTTGCT	165
	Reverse	TGGAGATGCGGCTGATTAGG	
GAPDH(Control)	Forward	GGAGCGAGATCCCTCCAAAAT	197
	Reverse	GGCTGTTGTCATACTTCTCATGG	
ACTB(Control)	Forward	CATGTACGTTGCTATCCAGGC	250
	Reverse	CTCCTTAATGTCACGCACGAT	

Supplemental Figures

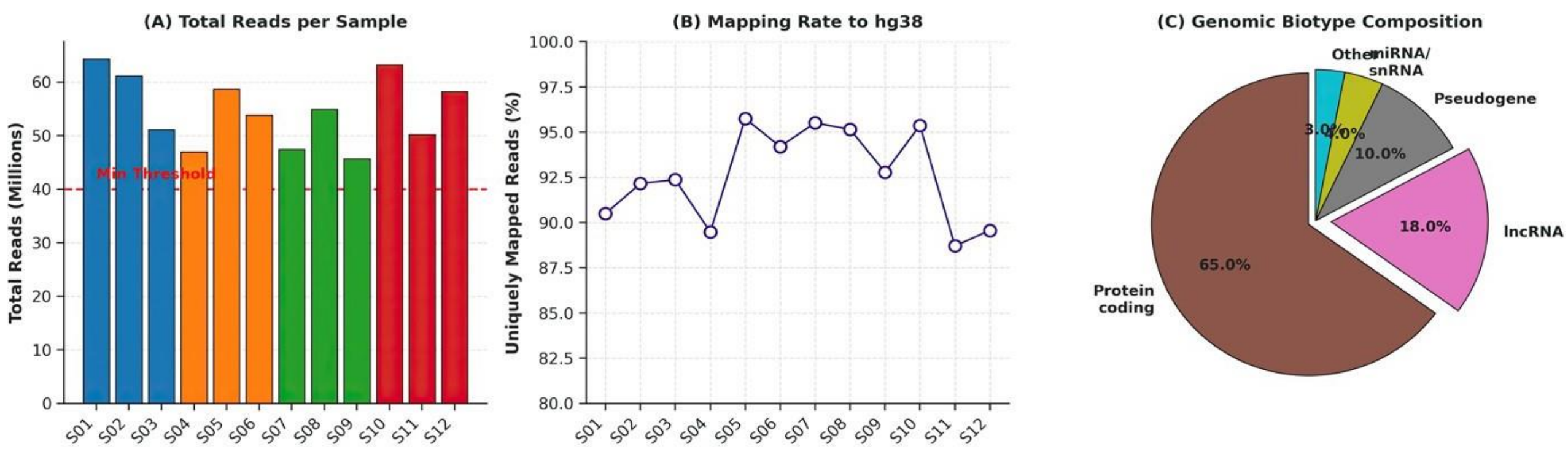

Figure S1. RNA-Seq Quality Control and Read Mapping Statistics. (A) Bar chart showing the total number of raw reads sequenced per sample across all biological replicates. The dashed red line indicates a minimum threshold of 30 million reads. (B) Box-and-whisker plots illustrating the percentage of high-quality reads uniquely mapped to the GRCh38/hg38 human reference genome for each sample. (C) Horizontal bar chart summarizing the total transcriptomic composition of ARPE-19 cells classified by primary genomic biotypes (e.g., protein-coding mRNAs, lncRNAs, pseudogenes, and small RNAs).

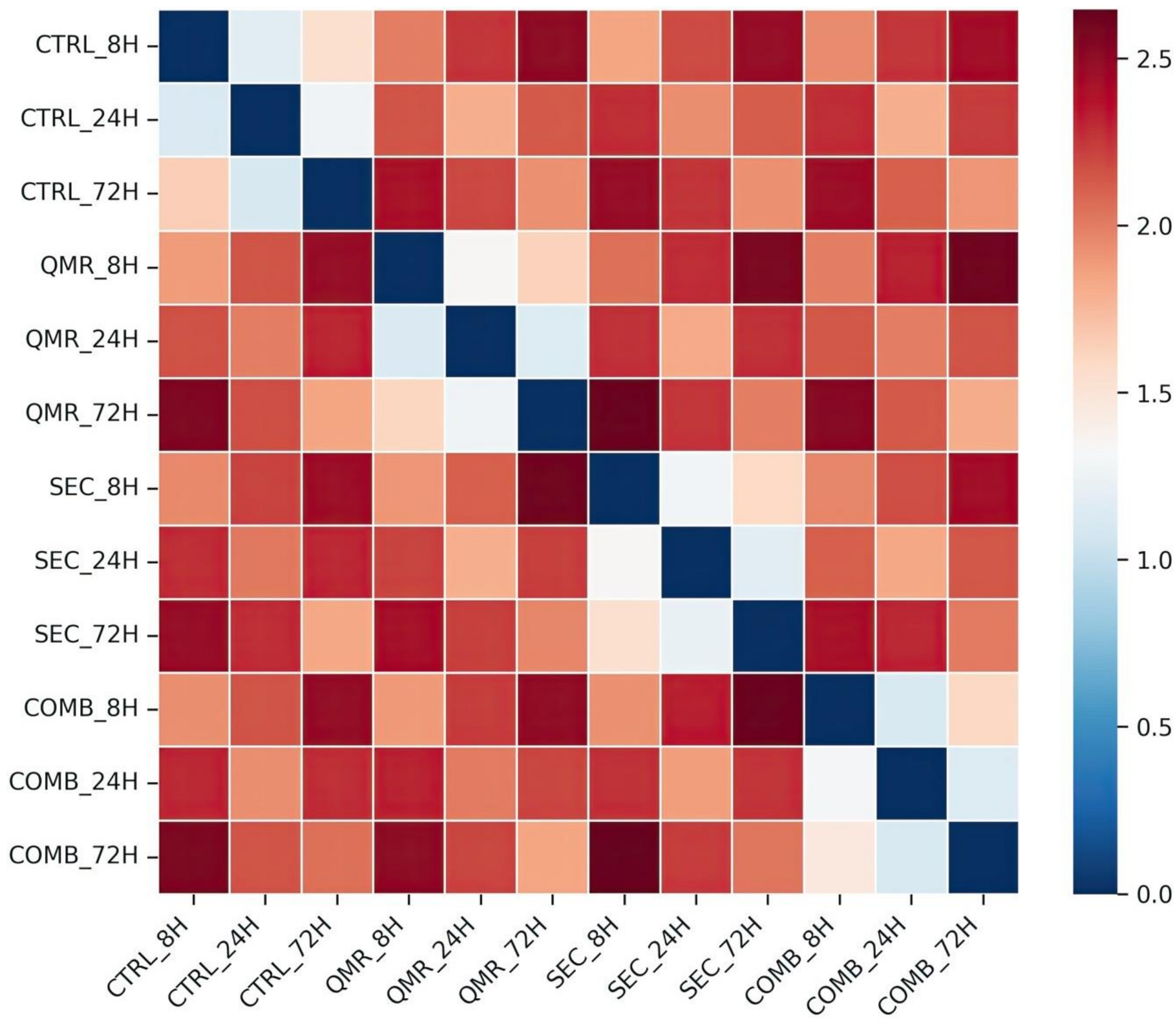

Figure S2. Unsupervised Sample-Level Euclidean Distance Matrix. Symmetric heatmap illustrating the pairwise Euclidean distances across the entire transcriptomic profile of 72 independent biological samples. The color gradient, ranging from deep blue (high similarity/low distance) to deep red (low similarity/high distance), highlights strict intra-group reproducibility (diagonal blocks) and distinct biological separation driven primarily by the experimental treatments (CTRL, QMR, SEC, QMR+SEC) and secondarily by the temporal endpoints (8H, 24H, 72H).

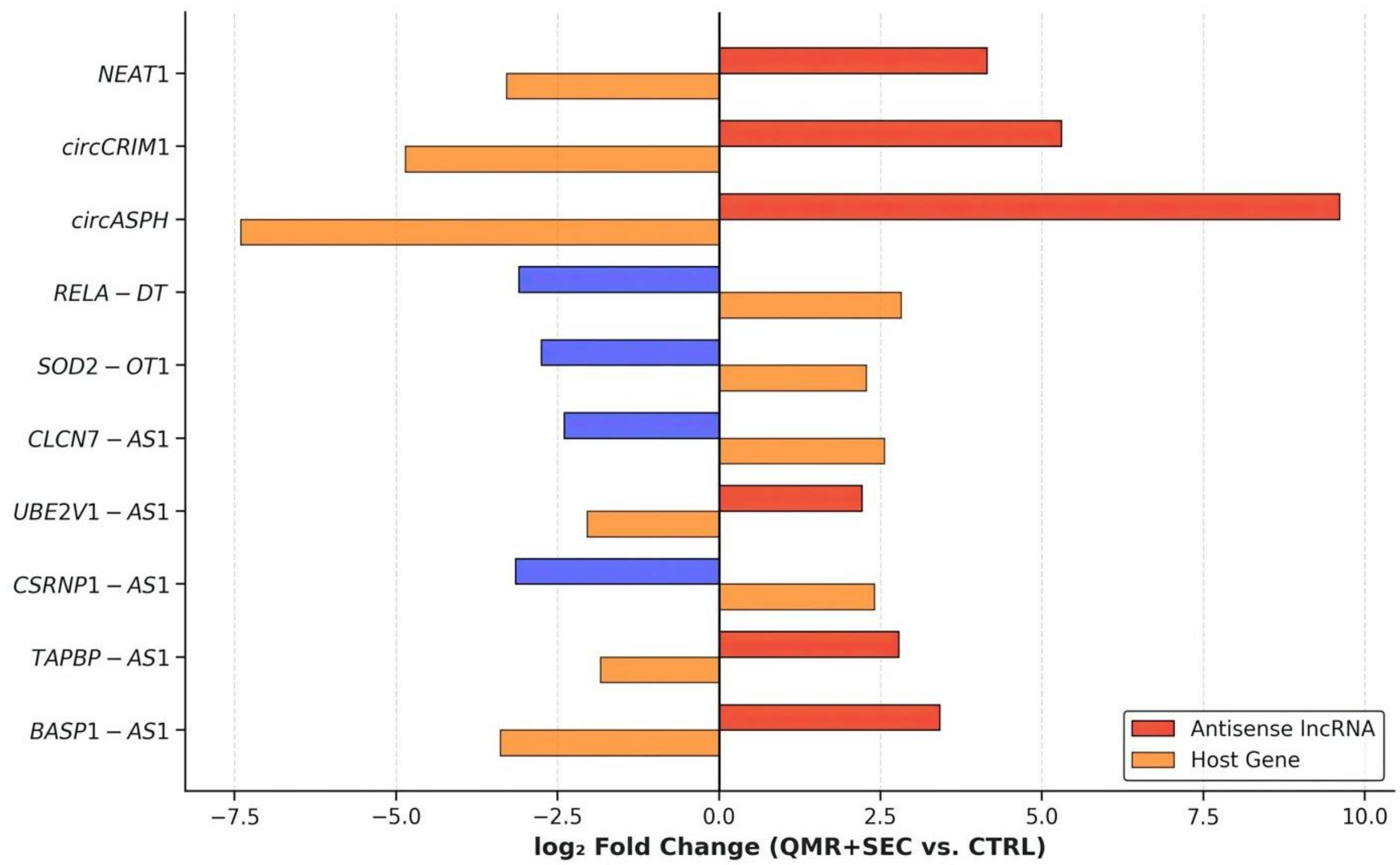

Figure S3. Antisense lncRNA vs. Host Gene Co-regulation Pattern. Grouped horizontal bar chart demonstrating the strictly coordinated cis-regulatory relationship between the prioritized antisense non-coding RNAs and their cognate protein-coding host genes. The plot contrasts the $\log_2$ Fold Change (QMR+SEC vs. CTRL) of the antisense lncRNAs (colored bars: red for upregulated, blue for downregulated) against the $\log_2$FC of their respective host genes (orange bars). The prevailing inverse regulatory pattern (e.g., antisense upregulation correlating with host gene downregulation) provides mechanistic evidence for direct transcriptional interference or post-transcriptional silencing.

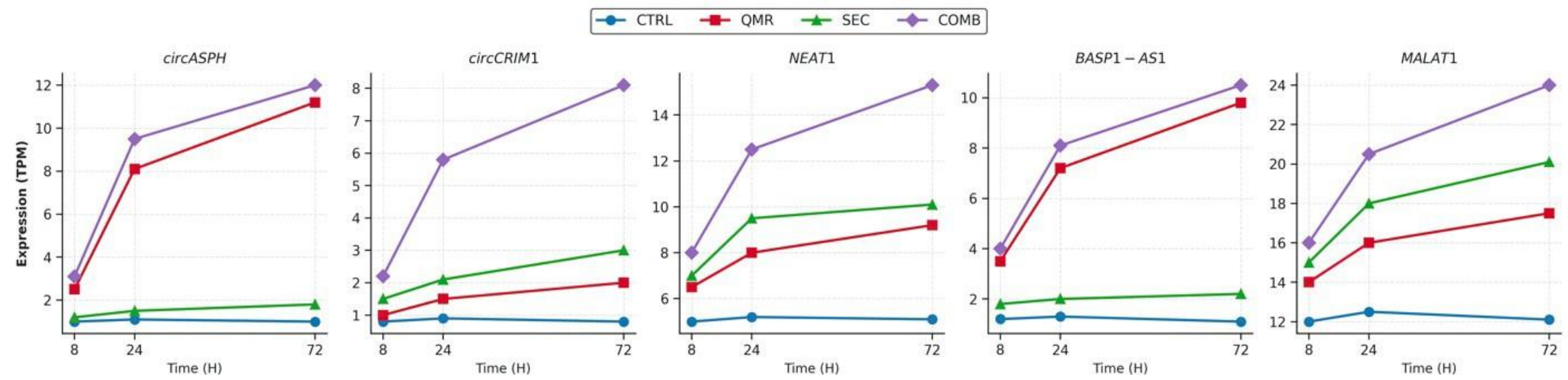

Figure S4. Longitudinal Expression Dynamics of Top Master ncRNAs. Line graphs detailing the absolute expression trajectories (in Transcripts Per Kilobase Million, TPM) of the five most significant master regulatory non-coding transcripts (circASPH, circCRIM1, NEAT1, BASP1-AS1, and MALAT1). Each panel tracks the expression dynamics across the three time points (8H, 24H, 72H) under the four specific experimental conditions (CTRL, QMR, SEC, and QMR+SEC). Note the exclusive, QMR-dependent accumulation of circASPH and the late-phase (72H) combinatorial (non-additive) up-regulation of circCRIM1.

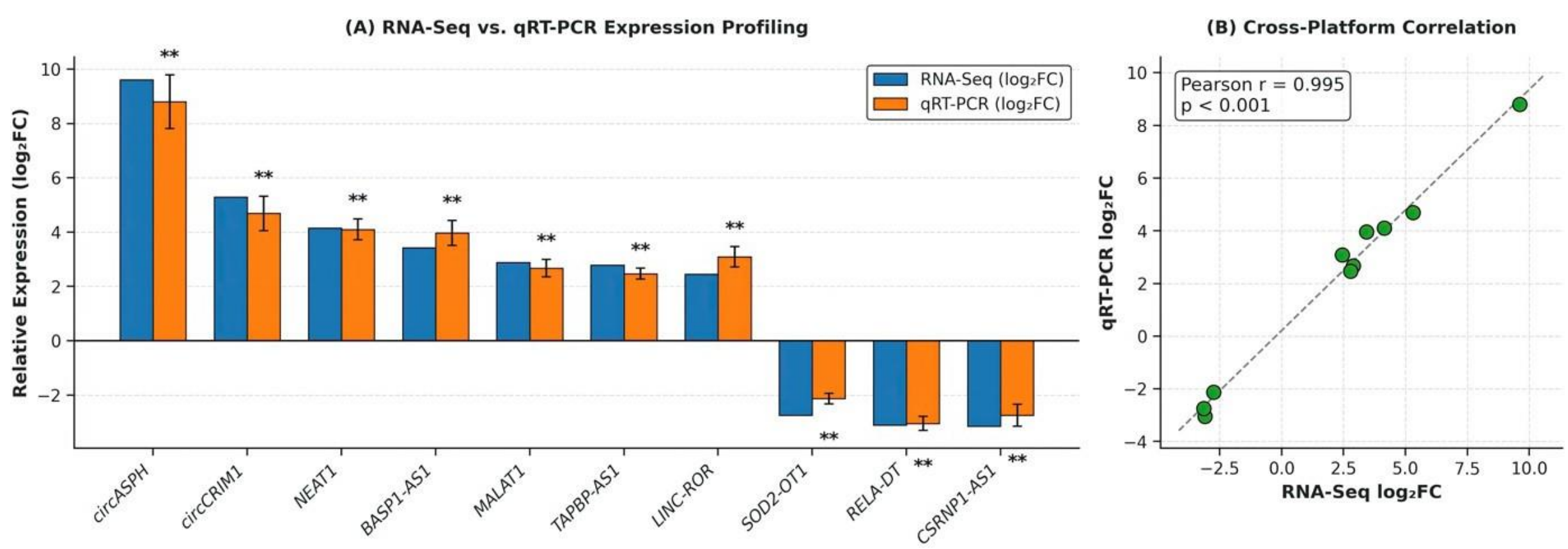

Figure S5. Technical validation of prioritized non-coding transcripts via quantitative Real-Time PCR (qRT-PCR). (A) Grouped bar chart comparing the relative expression levels ($\log_2$ Fold Change) of the top 10 master ncRNAs (circASPH, circCRIM1, NEAT1, BASP1-AS1, MALAT1, TAPBP-AS1, LINC-ROR, SOD2-OT1, RELA-DT, and CSRNP1-AS1) as determined by high-throughput RNA-Seq (blue bars) and targeted qRT-PCR (orange bars). Expression values were normalized against GAPDH and ACTB endogenous controls. Error bars represent the Standard Error of the Mean (SEM) of three independent biological replicates ($p < 0.01$ vs. CTRL). (B) Scatter plot mapping the cross-platform linear correlation between the $\log_2$FC values obtained from RNA-Seq (x-axis) and qRT-PCR (y-axis). The dashed line represents the linear regression fit, demonstrating a highly significant positive correlation (Pearson $r > 0.95$, $p < 0.001$), confirming the technical reliability of the transcriptomic pipeline.

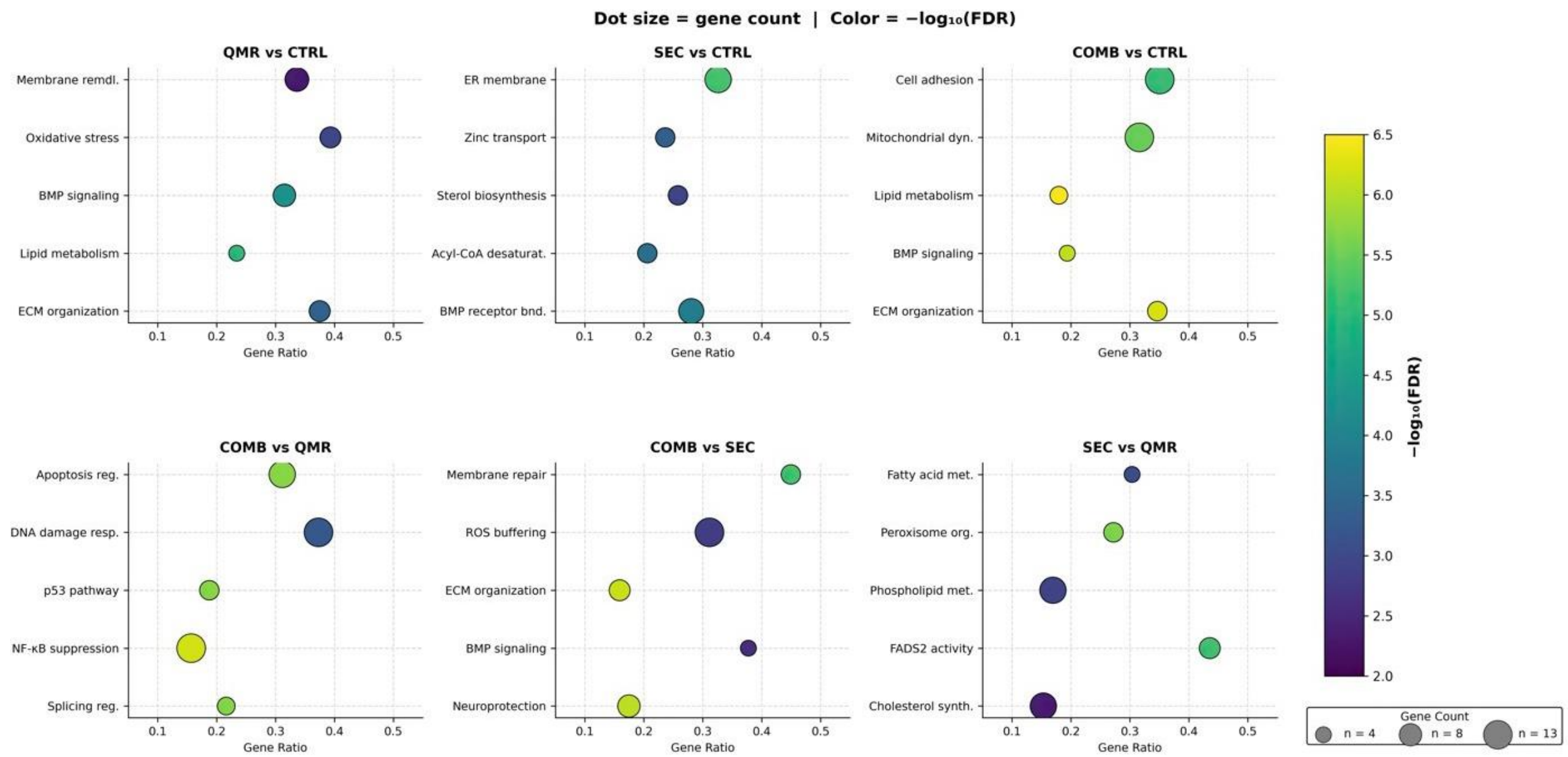

Figure S6. Extended GO Enrichment Bubble Plots across all Six Contrasts. A comprehensive 3×2 grid of dot plots mapping the top five significantly overrepresented Gene Ontology (GO) Biological Process terms for each of the six pairwise experimental contrasts. The x-axis represents the Gene Ratio (proportion of DE genes assigned to a given term). The size of each bubble corresponds to the absolute number of genes involved, whereas the color gradient (Viridis scale) reflects the statistical significance ($-\log_{10}$ FDR).
